# Functional enrichment analysis of LYSET and identification of related hub gene signatures as novel biomarkers to predict prognosis and immune infiltration status of clear cell renal cell carcinoma

**DOI:** 10.1007/s00432-023-05280-2

**Published:** 2023-09-23

**Authors:** Yuxing Chen, Jinhang He, Tian Jin, Ye Zhang, Yunsheng Ou

**Affiliations:** 1 Department of Orthopedics, The First Affiliated Hospital of Chongqing Medical University, Chongqing, China; 2 First Clinical Medical College, Chongqing Medical University, Chongqing, China; 3 Department of Urology, The First Affiliated Hospital of Chongqing Medical University, Chongqing, China; 4 Orthopedic Laboratory of Chongqing Medical University, Chongqing, China

**Keywords:** Amino acid metabolism reprogramming, Prognosis, Tumor immune microenvironment, Pathways, Immunotherapy, ccRCC, Lysosomal enzyme trafficking factor (LYSET)

## Abstract

**Purpose:**

The latest research shows that the lysosomal enzyme trafficking factor (LYSET) encoded by TMEM251 is a key regulator of the amino acid metabolism reprogramming (AAMR) and related pathways significantly correlate with the progression of some tumors. The purpose of this study was to explore the potential pathways of the TMEM251 in clear cell renal cell carcinoma (ccRCC) and establish related predictive models based on the hub genes in these pathways for prognosis and tumor immune microenvironment (TIME).

**Methods:**

We obtained mRNA expression data and clinical information of ccRCC samples from The Cancer Genome Atlas (TCGA), E-MATE-1980, and immunotherapy cohorts. Single-cell sequencing data (GSE152938) were downloaded from the Gene Expression Omnibus (GEO) database. We explored biological pathways of the LYSET by Gene Ontology (GO) and Kyoto Encyclopedia of Genes and Genomes (KEGG) analyses of TMEM251-coexpression genes. The correlation of LYSET-related pathways with the prognosis was conducted by Gene Set Variation Analysis (GSVA) and unsupervised cluster analysis. The least absolute shrinkage and selection operator (LASSO) and Cox regression were used to identify hub prognostic genes and construct the risk score. Immune infiltration analysis was conducted by CIBERSORTx and Tumor Immune Estimation Resource (TIMER) databases. The predictive value of the risk score and hub prognostic genes on immunotherapy responsiveness was analyzed through the tumor mutation burden (TMB) score, immune checkpoint expression, and survival analysis. Immunohistochemistry (IHC) was finally used to verify the expressions of hub prognostic genes.

**Results:**

The TMEM251 was found to be significantly correlated with some AAMR pathways. AAGAB, ENTR1, SCYL2, and WDR72 in LYSET-related pathways were finally identified to construct a risk score model. Immune infiltration analysis showed that LYSET-related gene signatures significantly influenced the infiltration of some vital immune cells such as CD4 + cells, NK cells, M2 macrophages, and so on. In addition, the constructed risk score was found to be positively correlated with TMB and some common immune checkpoint expressions. Different predictive values of these signatures for Nivolumab therapy responsiveness were also uncovered in immunotherapy cohorts. Finally, based on single-cell sequencing analysis, the TMEM251 and the hub gene signatures were found to be expressed in tumor cells and some immune cells. Interestingly, IHC verification showed a potential dual role of four hub genes in ccRCC progression.

**Conclusion:**

The novel predictive biomarkers we built may benefit clinical decision-making for ccRCC. Our study may provide some evidence that LYSET-related gene signatures could be novel potential targets for treating ccRCC and improving immunotherapy efficacy. Our nomogram might be beneficial to clinical choices, but the results need more experimental verifications in the future.

**Supplementary Information:**

The online version contains supplementary material available at 10.1007/s00432-023-05280-2.

## Introduction

Renal carcinoma is a kind of common urinary cancer. There were 431,288 new cases and 179,368 cases died because of it in 2020–2021 (Sung et al. 2021). Clear cell renal cell carcinoma (ccRCC) is the most common pathological type and accounts for most deaths of cancers in the kidney (Hsieh et al. 2017). Renal carcinoma patients in the early stage have an 80–90% survival rate at 5 years but have a poor prognosis in the advanced stage (Campbell et al. 2017). Targeted therapy is one of the main modalities for the treatment of advanced ccRCC. However, most patients develop resistance over time (Capitanio and Montorsi 2016). Immunotherapy is another effective choice for advanced ccRCC because of overexpressed PD-L1 in nearly 30% of patients (Rodriguez-Vida et al. 2017), but some patients can't benefit from immunotherapy (Rini et al. 2019). Therefore, accurate predictive models for the prognosis and the immunotherapy responsiveness of ccRCC are urgent to be constructed.

Cancer metabolism reprogramming is a process of remodeling or reprogramming metabolism pathways involved in biosynthesis, nutrient uptake, and redox balance. Metabolism reprogramming helps cancer cells adapt to high energy requirements and maintain rapid proliferation (Chakraborty et al. 2021). In all tumor metabolic pathways, amino acid metabolism is a crucial part and its reprogramming has been revealed to promote tumor progression through influencing gene expression, cell state, and tumor immune microenvironment (TIME) in prostate cancer, colorectal cancer, breast cancer, renal cell cancer, and so on (Chakraborty et al. 2021; Li et al. 2022; Ahmad et al. 2021; Chen et al. 2022; Corchado-Cobos et al. 2022; Kamphorst et al. 2015; Raggi et al. 2022). Meanwhile, many studies have shown that extra intracellular and extracellular protein usage mainly by enhanced pinocytosis, lysosomal protein degradation, and even proteasome-mediated degradation is a vital pathway of amino acid metabolism reprogramming (AAMR) for cancer cells, and it mediates the tolerance of tumor cells to amino acid deficiency in the nutrient-deficient tumor microenvironment (TME). In all, obtaining amino acid supplements by degrading proteins is essential for cancer cells’ survival in nutrient-deficient TME (Corchado-Cobos et al. 2022; Martinez-Reyes and Chandel 2021; Rousseau and Bertolotti 2018; Suraweera et al. 2012; Kwon and Ciechanover 2017).

Several latest studies have found that the lysosomal enzyme trafficking factor (LYSET) encoded by the TMEM251 gene is a key protein for normal components and functions of the lysosome. LYSET, as the first regulator of mannose 6-phosphate modification, can guarantee normal intracellular sorting of most lysosome enzymes (Richards et al. 2022; Zhang et al. 2022a). Moreover, the latest experiment reported in the science journal shows the progressions of cancers without LYSET were inhibited because of failure in lysosomal protein degradation for amino acid supplements in nutrient-deficient TME but was not influenced in amino acid-rich conditions (Pechincha et al. 2022). The above results indicate that LYSET is a key factor for cancer cells to tolerate amino acid-deficient TME. A real TME is usually characterized by reduced amino acid levels (Li et al. 2021). Therefore, LYSET plays a vital role in tumor growth and its deficiency may destroy the ability of cancer cells to tolerate amino acid deficiency.

**Fig. 1:**
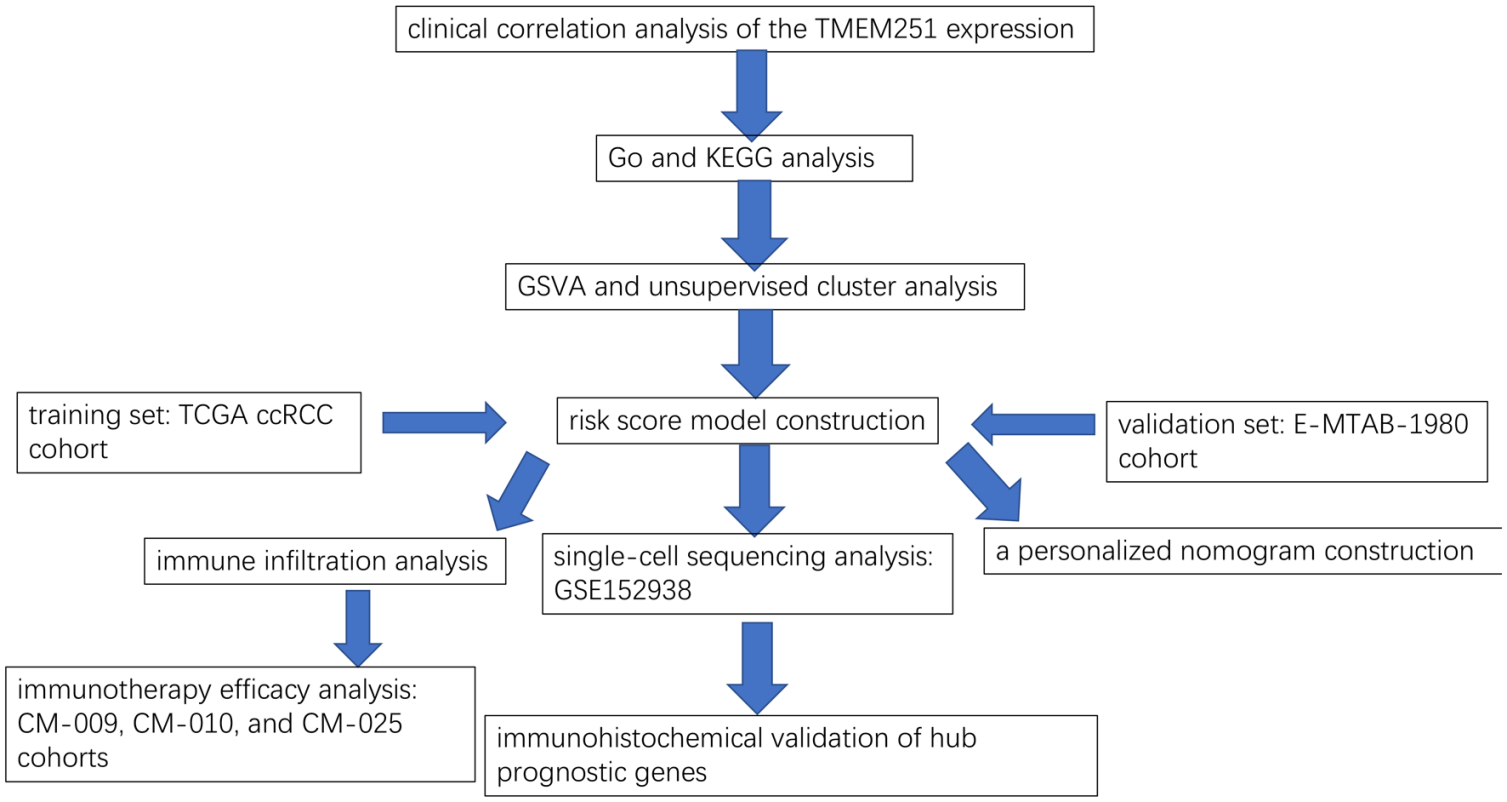
The whole analysis process of this study

AAMR is a typical feature of ccRCC cells, and it can promote tumor progression by influencing the TIME. Thus, the LYSET shows potential prognostic and treatment values in ccRCC (Chakraborty et al. 2021; Wettersten et al. 2017). In this study, we aimed to explore the biological pathways and prognostic values of the LYSET in ccRCC through bioinformatics analysis. Moreover, we tried to construct risk and predictive models based on several key genes related to the LYSET and evaluated the models through external validation. To reveal possible mechanisms of the prognostic function of the risk model, we also explored connections between it and the TIME in ccRCC. Finally, we explored predictive values of the risk model for immunotherapy responsiveness based on the associations between the risk score and the TIME. Our analysis results may promote a deeper understanding of the roles of LYSET in ccRCC and be beneficial to clinical choices. The analysis process of this study is shown in Fig. 1.

## Materials and methods

### Acquisition and processing of data

The transcripts per million (TPM) mRNA expression dataset and associated clinical information of 540 cancer samples and 72 normal samples of ccRCC were downloaded from The Cancer Genome Atlas (TCGA) cohort (https://portal.gdc.cancer.gov) for an experimental set. Gene mutation data from 330 out of the 540 samples were also obtained from the TCGA cohort. Then the duplicate genes were removed by R software. Subsequently, the mRNA expression data from the same samples were averaged. Furthermore, the genes that were not expressed in all cancer samples were deleted. Finally, we got 532 cancer samples and 72 normal samples with respective 19,518 gene expressions. As an external validation set of the risk and the predictive models, the normalized gene expression data and associated clinical information of the E-MTAB-1980 cohort (101 cancer samples) were downloaded from the ArrayExpress (https://www.ebi.ac.uk/biostudies/arrayexpress). Protein–Protein Interaction Networks (PPI) were obtained from the String database (https://cn.string-db.org). The CIBERSORTx (http://cibersortx.stanford.edu) and Tumor Immune Estimation Resource (TIMER) databases (timer.cistrome.org) were used for immune infiltration analysis. CM-009 (NCT01358721), CM-010 (NCT01354431), and CM-025 (NCT01668784) immune checkpoint inhibitor (ICI) therapy cohorts were downloaded from a previous article (Braun et al. 2020).

### Gene Ontology (GO), Kyoto Encyclopedia of Genes and Genomes (KEGG), and Protein–Protein Interaction Networks (PPI) analyses of TMEM251-coexpression genes

A heatmap between TMEM251 and clinicopathological factors in cancer samples was drawn by the heatmap R package in the experimental group. The connections between TMEM251 expressions and some clinicopathological factors (T_stage, M_stage, N_stage, stage, age, gender, race, and prior malignancy history) were described by the ggplot2 R package.

The Pearson correlation analysis between TMEM251 and other genes was performed by the cor function in R software. Pearson correlation coefficient (*R*) > 0.4 and statistical significance (*P* value) < 0.05 were the criteria for identifying the genes that were highly positively correlated with TMEM251.

Gene Ontology (GO) and Kyoto Encyclopedia of Genes and Genomes (KEGG) methods are frequently used to assess the biological functions and the signaling pathways of polygenes. In our study, GO and KEGG methods were used by the DAVID tool (http://david.ncifcrf.gov) to analyze the biological functions and the signaling pathways of TMEM251-coexpression genes in the experimental group. The top five enriched gene sets and 20 enriched gene sets possibly related to AAMR were visualized by the ggplot2 R package and the SangerBox online tool (SangerBox.com). Finally, the 20 enriched gene sets possibly related to AAMR were identified, and 310 genes of these gene sets were obtained. PPI was used to uncover potential protein-level connections among the 310 genes.

### Gene Set Variation Analysis (GSVA) of the 20 enriched gene sets and unsupervised cluster analysis

GSVA was conducted in the experimental group to calculate the respective enrichment score of the 20 enriched gene sets in GO and KEGG results by the GSVA R package (Hänzelmann et al. 2013). A heatmap was used to describe the associations between enrichment scores and TMEM251 expression. Meanwhile, batch Pearson correlation analysis between TMEM251 and enrichment scores was performed by R software and was visualized in the bar chart. Finally, associations between enrichment scores and some clinicopathological factors (tumor stage, T stage, N stage, and M stage) were visualized into boxplots by the ggplot2 R package.

Unsupervised cluster analysis is usually performed to divide samples into different subgroups according to some internal characteristics. In this study, to furtherly elucidate the impacts of LYSET protein, related biological process, and related pathways on the prognosis of ccRCC, we conducted unsupervised cluster analysis three times in the experimental group according to TMEM251-coexpression genes, genes of the 20 enriched gene sets, and GSVA enrichment scores of the 20 enriched gene sets by K-means method (maxK = 9, reps = 1000, pItem = 0.8, distance = Euclidean) using the ConsensuClusterPlus R package (Wilkerson and Hayes 2010). Kaplan–Meier (KM) curves based on cluster groups were drawn to show different prognostic outcomes among clusters in ccRCC.

### Differential gene expression analysis and identification of prognostic genes

The mRNA expression data were transformed by log2(*x* + 1) before analysis. The differential gene expression analysis was performed by the Limma R package, and differentially expressed genes (DEGs) were obtained according to the cutoff criterion of |Log2FC|> 0.5 and *P* value < 0.05 (Ritchie et al. 2015). Subsequently, TMEM251-coexpression genes associated with prognosis were determined from the intersection of the 20 enriched gene sets related to AAMR in GO and KEGG results and DEGs between cancer and normal samples in the experimental group.

### Construction of a risk score model and analysis of related clinical value

Four cancer samples that had not undergone follow-up inquiry (overall survival = 0) were deleted in the experimental group. Based on the data of the experimental group, the least absolute shrinkage and selection operator (LASSO) regression analysis was used to screen key genes in TMEM251-coexpression genes associated with the prognosis and calculate the corresponding lambda value by the glmnet and survival R package (Friedman et al. 2010). The univariate Cox regression analysis was performed to furtherly determine hub genes. Connections between hub gene expressions and some clinicopathological factors were described by violin plots. Risk scores of 528 experimental group samples and 101 validation group samples were calculated as follows:

$${\text{risk~score~}} = \left( {{\text{Exprgene}} - 1 \times \lambda {\text{gene}} - 1} \right) + \left( {{\text{Exprgene}} - 2 \times \lambda {\text{gene}} - 2} \right) + \cdots + \left( {{\text{Exprgene}} - n \times \lambda {\text{gene}} - n} \right),$$

where Exprgene is the expression level of the hub genes and *λ*gene is the corresponding coefficient value in univariate cox regression analysis.

Associations between the risk score and clinicopathological factors were visualized by the ggplot2 R package. Moreover, the receiver operating characteristic (ROC) curve was drawn, and area under curve (AUC) was calculated by the timeROC and ggplot2 R package to check the discrimination ability of the risk score to predict the prognosis.

### Kaplan–Meier (KM) curves for different risk groups and construction of an predictive model

The samples of the experimental (528 samples) and the validation groups (101 samples) were both divided into low-risk and high-risk groups according to the respective median cutoff of risk scores. We used the survminer, survival, ggplot2, and ggpubr R packages to draw KM curves based on the risk group. Subsequently, based on the data of the experimental group, 282 samples with incomplete clinicopathological information were excluded, and we incorporated nine clinicopathological factors including the risk score to perform stepwise multivariate Cox regression analysis based on the Akaike information criterion (AIC) index. According to the minimum AIC index, five factors were identified. Subsequently, we excluded factors with no statistical significance. Finally, four clinicopathological factors were determined to construct a multivariate Cox proportional hazard regression predictive model and draw a nomogram by the survival and rms R package in the experimental group. Meanwhile, calibrate curves in experimental and validation groups were drawn to evaluate the accuracy of the model and the concordance index (C-index) values were calculated to evaluate the discrimination ability of the model.

### Gene Set Enrichment Analysis (GSEA) between different risk groups

We performed GSEA between different risk groups according to official GO and KEGG gene sets to uncover potential molecular mechanisms (Subramanian et al. 2005). Initial analysis results were obtained from GSEA analysis software and we used NOM *P* val < 0.05 and FDR < 0.25 as the criterion for screening final significantly enriched GO and KEGG pathways. Some pathways possibly related to AAMR were found to be significantly enriched in the high-risk group, and several most significant pathways of them were shown in this study.

### Immune infiltration analysis

Immune cell infiltration scores were calculated in the TCGA cohort by the CIBERSORT method on the CIBERSORTx website (http://cibersortx.stanford.edu) (Newman et al. 2019). A proportion plot was used to show immune cell infiltration ratios. The risk scores of 532 experimental group samples were calculated according to the formula mentioned in the previous section and all samples were divided into low-risk group and high-risk group according to the median cutoff. A heatmap was drawn to show relationships between the risk score and immune cell infiltration scores by the heatmap R package. Meanwhile, we drew a boxplot to describe different immune cell infiltration scores between two risk groups and used the batch Spearman correlation method by the cor function in R to analyze the correlations between risk scores and infiltration scores. The correlations were visualized by the circlize R package and the Photoshop software. To furtherly explore the impacts of the hub risk genes on TIME, Spearman correlation analysis results between these genes and nine common immune cells infiltration scores were also obtained from the TIMER database (timer.cistrome.org).

### Tumor mutation burden (TMB) analysis

The risk scores of 330 samples with gene mutation information were calculated according to the previous formula. Meanwhile, the samples were divided into low-risk and high-risk groups according to the median cutoff. The TMB score of every sample was calculated by the maftools R package (Mayakonda et al. 2018). TMB score differences between risk groups were visualized by the ggpubr and ggplot2 R packages. The Pearson correlation analysis between the TMB score and the risk score was performed by the cor function in R.

### Correlation analysis between the risk score and immunotherapy efficacy

We used the stat_cor function in R to perform batch Pearson correlation analyses between the risk score and common immune checkpoint genes in the experimental group. The ggplot2, reshape2, ggpubr, and patchwork R packages were used to visualize the results. The risk score of every patient in immunotherapy cohorts was calculated according to the previously mentioned formula and all samples were divided into low-risk and high-risk groups according to the median level. Meanwhile, samples were also divided into low-expression and high-expression groups according to the median expression level of hub genes. KM curves based on the risk groups and the hub gene expression groups were drawn to describe different overall survival rates in the immunotherapy cohort.

### Single-cell sequencing analysis

The Seurat R package was used to load the 10X sequencing data into R software (Stuart et al. 2019; Butler et al. 2018). The screening criteria for low-quality cells are as follows: 1. Those with identified genes < 200 or > 5000. 2. Those with a percentage of mitochondrial genes over 40% of total expressed genes. The low-quality cells were filtered out before the analysis. We chose the top 2000 highly variable genes for further principal component analysis (PCA). FindNeighbors and FindClusters functions were used for cell clustering based on 50 principal components. The cell clustering results were displayed in the uniform manifold approximation and projection (UMAP) plot. Every cluster was renamed based on some classical cell markers from the CellMarker 2.0 database (http://117.50.127.228/CellMarker/). Finally, the expressions of TMEM251, AAGAB, ENTR1, SCYL2, and WDR72 were identified in every cell cluster.

### Immunohistochemical staining of the four hub prognostic genes

First, we performed the differential mRNA expression analysis of the four hub prognostic genes and then the corresponding immunohistochemistry (IHC) staining results were downloaded from the Human Protein Atlas (HPA) database (Thul and Lindskog 2018) to verify their protein-level expressions. Subsequently, we validated the protein-level expressions of the four hub prognostic genes by IHC experiment. This study was approved by the Ethics Committee at the First Affiliated Hospital of Chongqing Medical University (approval No. 2020-049) and by all participants who provided their pathological tissues. Tumor and corresponding paracancerous tissue sections of ccRCC were collected in surgical treatment from the First Affiliated Hospital of Chongqing Medical University. We prepared slices according to the detailed steps in our previous study (Wang et al. 2022). After blocking, the anti-AAGAB, anti-ENTR1, anti-SCYL2, and anti-WDR72 antibodies were stained to sections. The remaining experimental steps were according to the detailed descriptions in our previous study (Wang et al. 2022). The SlideViewer software and KF-Viewer software were used to capture images.

### Statistical analyses

All statistical analyses in this study were carried out by R and SPSS software. The differences between two groups were compared by the Wilcoxon test and three or more groups were compared by the Kruskal–Wallis test. The statistical significance (*P* value) < 0.05 was thought to be significant.

## Results

### Clinical correlation analysis of TMEM251 expression

**Fig. 2:**
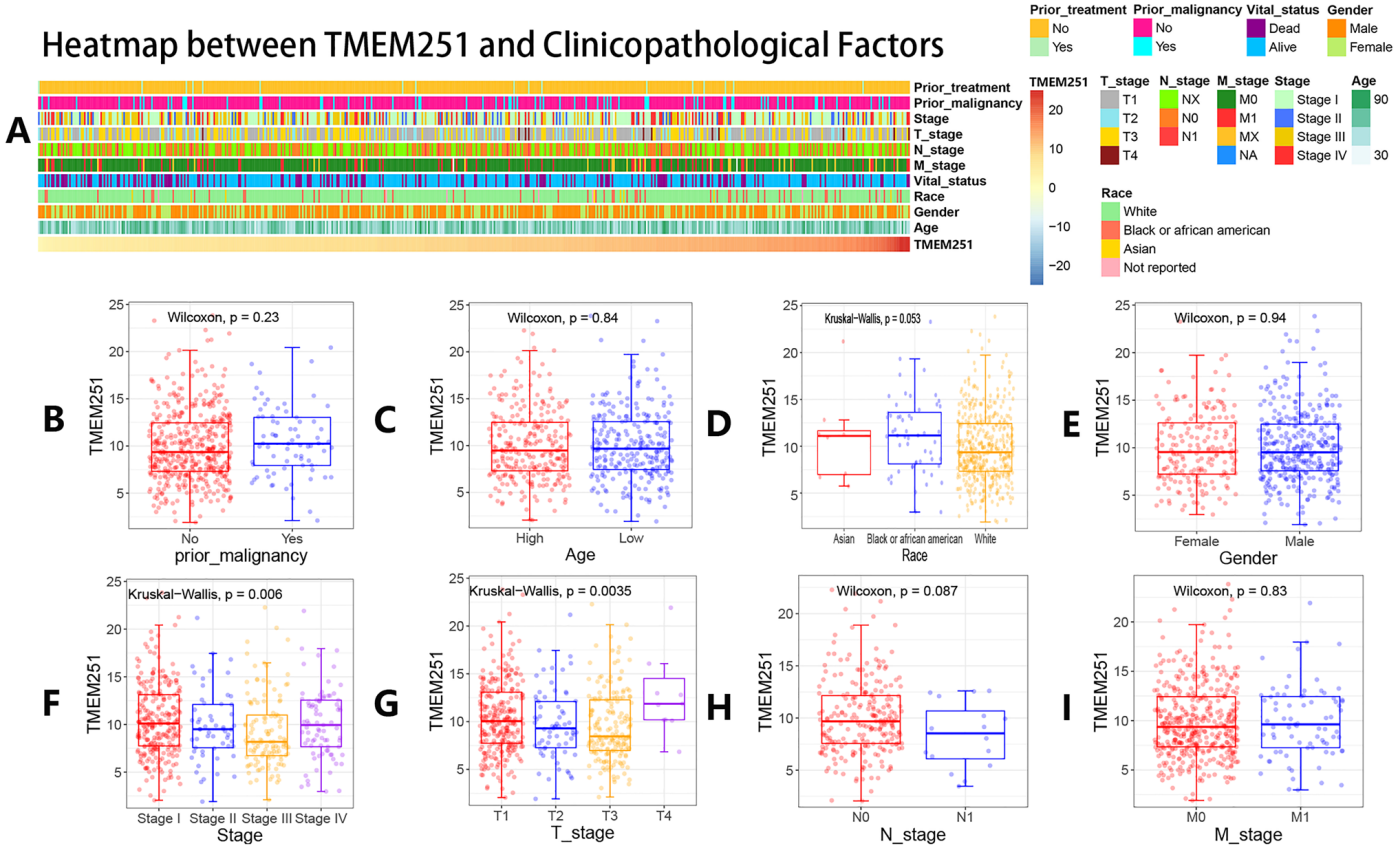
Associations between the TMEM251 expression and clinicopathological information. **A** Heatmap between the TMEM251 expression and clinicopathological factors. **B**–**I** Comparisons of TMEM251 expressions in different vital clinicopathological factors

The heatmap showed close connections between TMEM251 expression and clinicopathological factors in the experimental group (Fig. 2A). Furthermore, several boxplots were used to describe specific TMEM251 expression differences in some vital clinicopathological factors (stage, T stage, N stage, M stage, age, gender, race, and prior malignancy history) (Fig. 2B–I). Interestingly, the TMEM251 expression was found to be significantly negatively correlated with the tumor stage and T stage in the relatively early stage (stage I-III and T stage 1–3) of ccRCC. However, the TMEM251 expression was revealed to be increased in the advanced stage (stage IV and T stage 4) of ccRCC. The above results indicated that TMEM251 might play a dual role in ccRCC development and could be a potential marker for advanced ccRCC. These results might be associated with the dual role of the TMEM251-related lysosome-mediated autophagy and will be discussed in detail in the discussion part.

### Functional enrichment analysis of the TMEM251

**Fig. 3:**
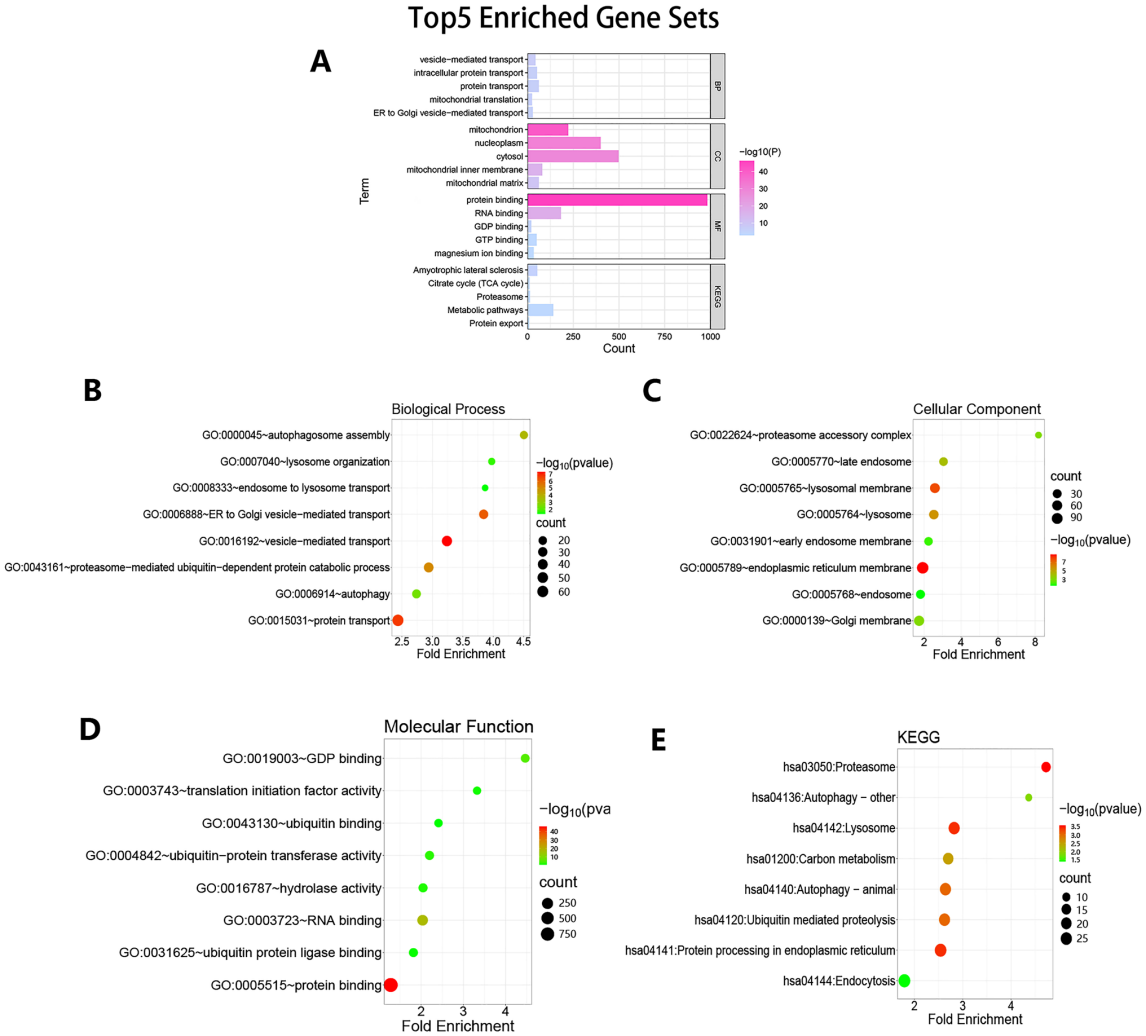
Biological functions and pathways analysis of 1234 TMEM251-coexpression genes. **A** Top five enriched gene sets in biological process (BP), cellular component (CC), molecular function (MF), and KEGG. **B**–**E** Some enriched gene sets related to the AAMR in BP, CC, MF, and KEGG analysis (some of them were not shown in the figure and they were displayed in supplementary materials)

We set correlation coefficient > 0.4 and *P* value < 0.05 as the selection criteria and finally identified 1234 TMEM251-coexpression genes based on the experimental group in the Pearson correlation analysis (Supplementary Table S1). Subsequently, GO and KEGG analyses for these genes were performed. The respective top five gene sets of analysis results are shown in Fig. 3A. The most enriched biological processes were vesicle-mediated transport, intracellular protein transport, and protein transport. Meanwhile, the citrate cycle (TCA cycle), proteasome, and metabolic pathways were found to be significantly enriched. Furthermore, some gene sets related to endocytosis, vesicle-related transport, lysosome-mediated autophagosome, lysosome-mediated extracellular protein degradation, and proteasome-mediated degradation processes were also found to be significantly enriched in biological process (BP), cellular component (CC), molecular function (MF), and associated signaling pathways (KEGG) results (Fig. 3B–E and Supplementary Table S2–5). These gene sets (20 in total) with 310 genes were selected as the research objects to perform subsequent analyses (Supplementary Table S2–6). PPI analysis showed protein-level interactions among these genes (Fig. S1). In conclusion, the above results indicated that TMEM251 might correlate with the AAMR especially extra usage of proteins for cancer cells in the starving state.

### Gene Set Variation Analysis (GSVA) of the 20 enriched gene sets

Enrichment scores of the 20 enriched gene sets were calculated in the experimental group, and the connections between scores and TMEM251 expression were visualized by the heatmap. Higher TMEM251 expression correlated with higher enrichment scores (Fig. S2). Moreover, the Pearson correlation analysis results (Fig. S2) indicated a positive correlation between TMEM251 expression and enrichment scores. Results shown in GSVA were consistent with that in GO and KEGG analyses.

**Fig. 4:**
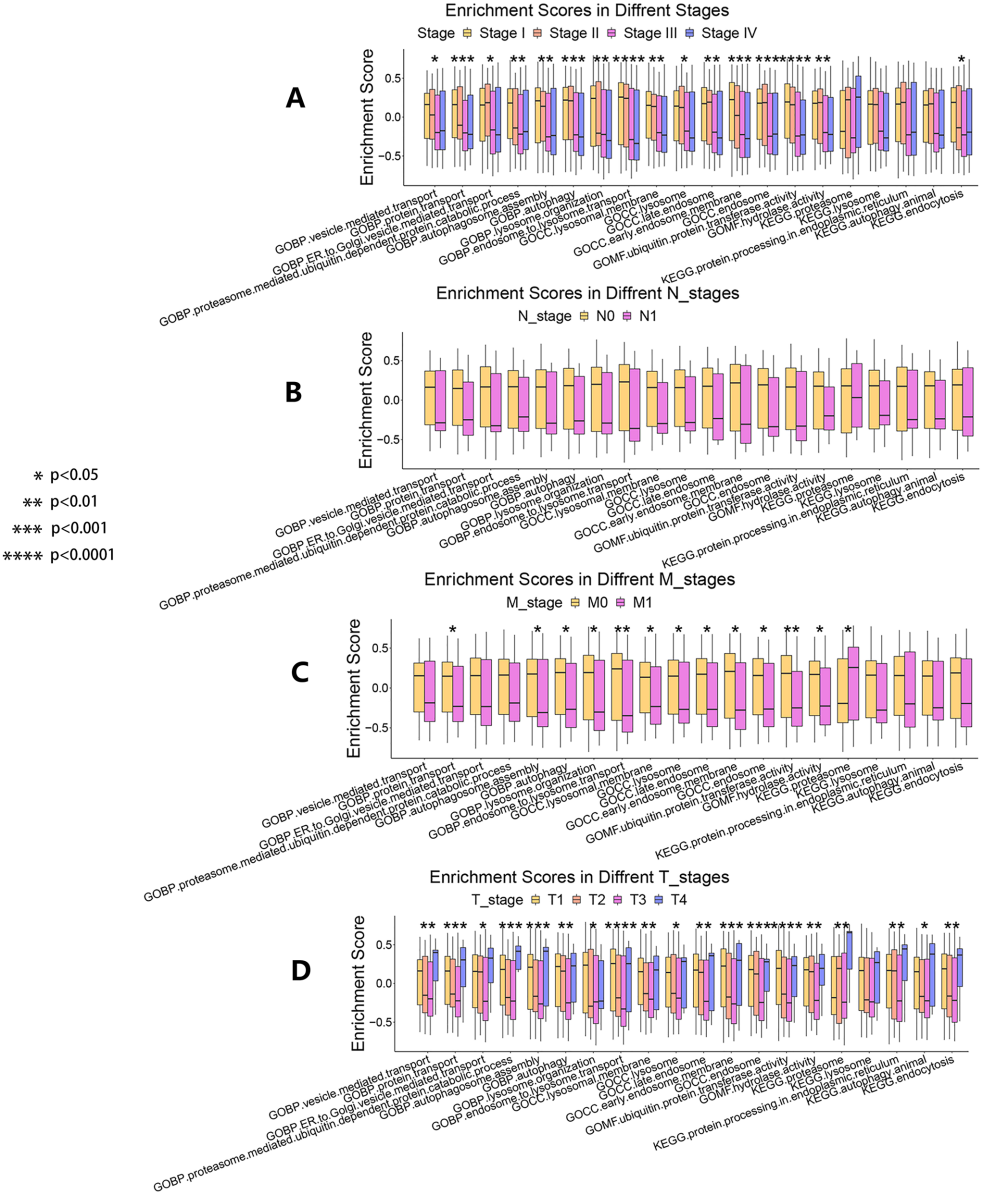
Connections between GSVA enrichment scores of the 20 AAMR-related enriched gene sets and tumor progression. **A** Enrichment scores comparisons based on the tumor stage. **B** Enrichment scores comparisons based on the N stage. **C** Enrichment scores comparisons based on the M stage. **D** Enrichment scores comparisons based on the T stage

In order to explore the impacts of the 20 enriched gene sets on the ccRCC progression, comparisons of the enrichment scores in different tumor stages, T stages, N stages, and M stages were conducted. The enrichment scores of some pathways were found to be significantly different in different tumor stages, but no pathways were significantly increased in the advanced tumor stages (Fig. 4A). Figure 4B shows that the enrichment scores had no significant differences in the N stage. For the M stage, higher KEGG proteasome enrichment scores were significantly connected with the M1 stage (Fig. 4C). Moreover, enrichment scores of almost all the 20 enriched gene sets were all significantly higher in the late T stages (Fig. 4D). Meanwhile, most pathways associated with the lysosome-mediated autophagy showed dual roles in ccRCC development because they were significantly negatively correlated with the T stage in T stage 1–3 but were increased in the T stage 4. This result was consistent with the clinical correlation analysis of the TMEM251 expression. In conclusion, all the above results indicated that the 20 enriched gene sets possibly influenced ccRCC progression by mainly affecting the tumor volume.

### Unsupervised cluster analysis

**Fig. 5:**
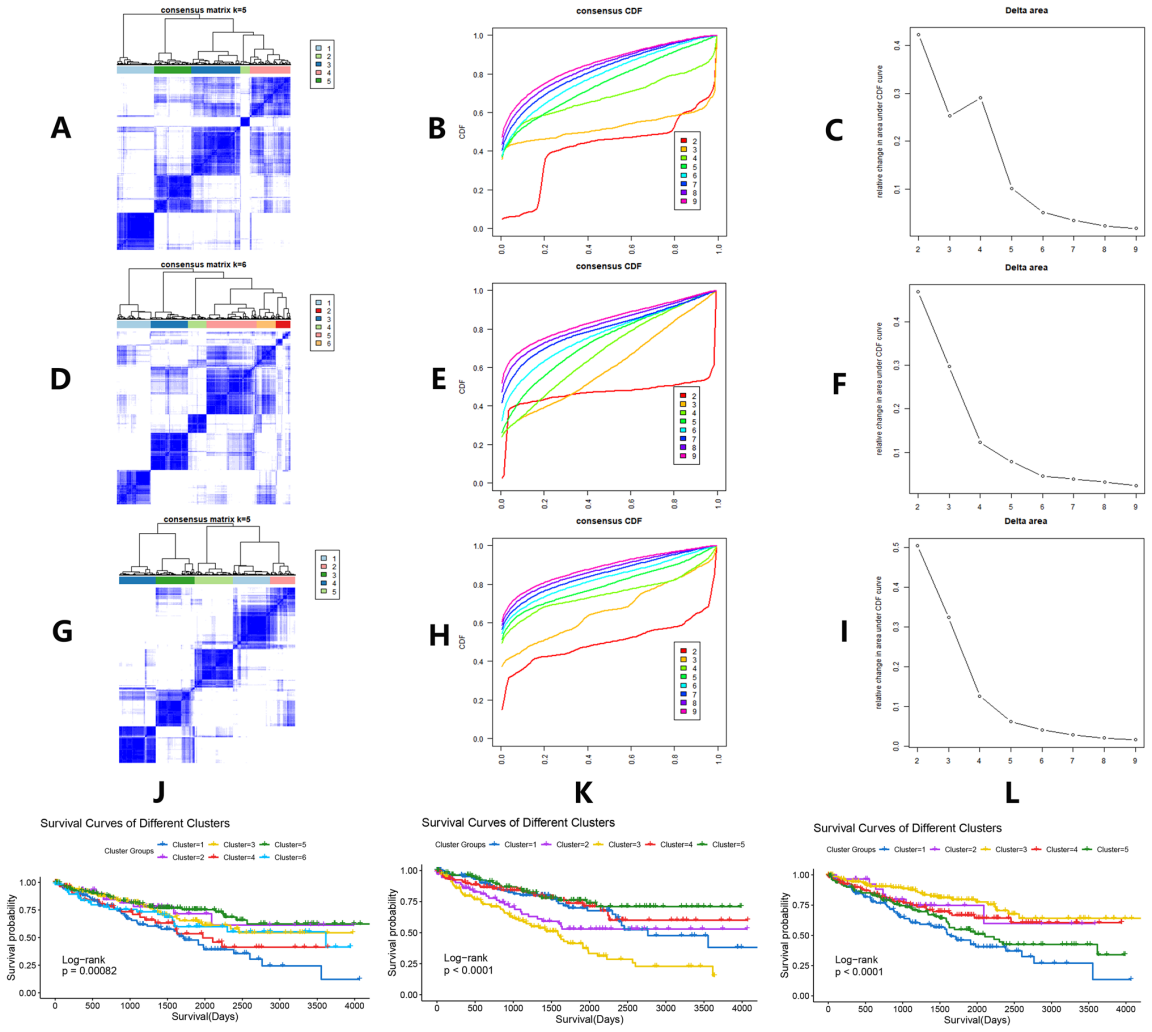
Unsupervised cluster analysis based on three patterns. **A**–**C** Cluster analysis based on 1234 TMEM251-coexpression gene expressions. **D**–**F** Cluster analysis based on 310 gene expressions of the 20 enriched gene sets related to AAMR. **G**–**I** Cluster analysis based on GSVA enrichment scores. **J** KM curves among clusters based on 1234 TMEM251-coexpression gene expressions. **K** KM curves among clusters based on 310 gene expressions of the 20 enriched gene sets related to AAMR. **L** KM curves among clusters based on GSVA enrichment scores

Unsupervised cluster analysis was performed three times according to 1234 TMEM251-coexpression genes (Fig. 5A–C), 310 genes in the 20 enriched AAMR-related gene sets (Fig. 5D–F), and GSVA enrichment scores (Fig. 5G–I). 5, 6, and 5 were determined as the best cluster group number. Analysis results were shown in Supplementary Table S7–9. KM curves were used to reveal their impacts on the ccRCC prognosis. Significantly different overall survival rates among clusters are shown in Fig. 5J–L. Cluster analysis results indicated that the LYSET and its related biological pathways genes could influence the prognosis of ccRCC.

### Differential gene expression analysis and determination of 55 prognostic genes

**Fig. 6:**
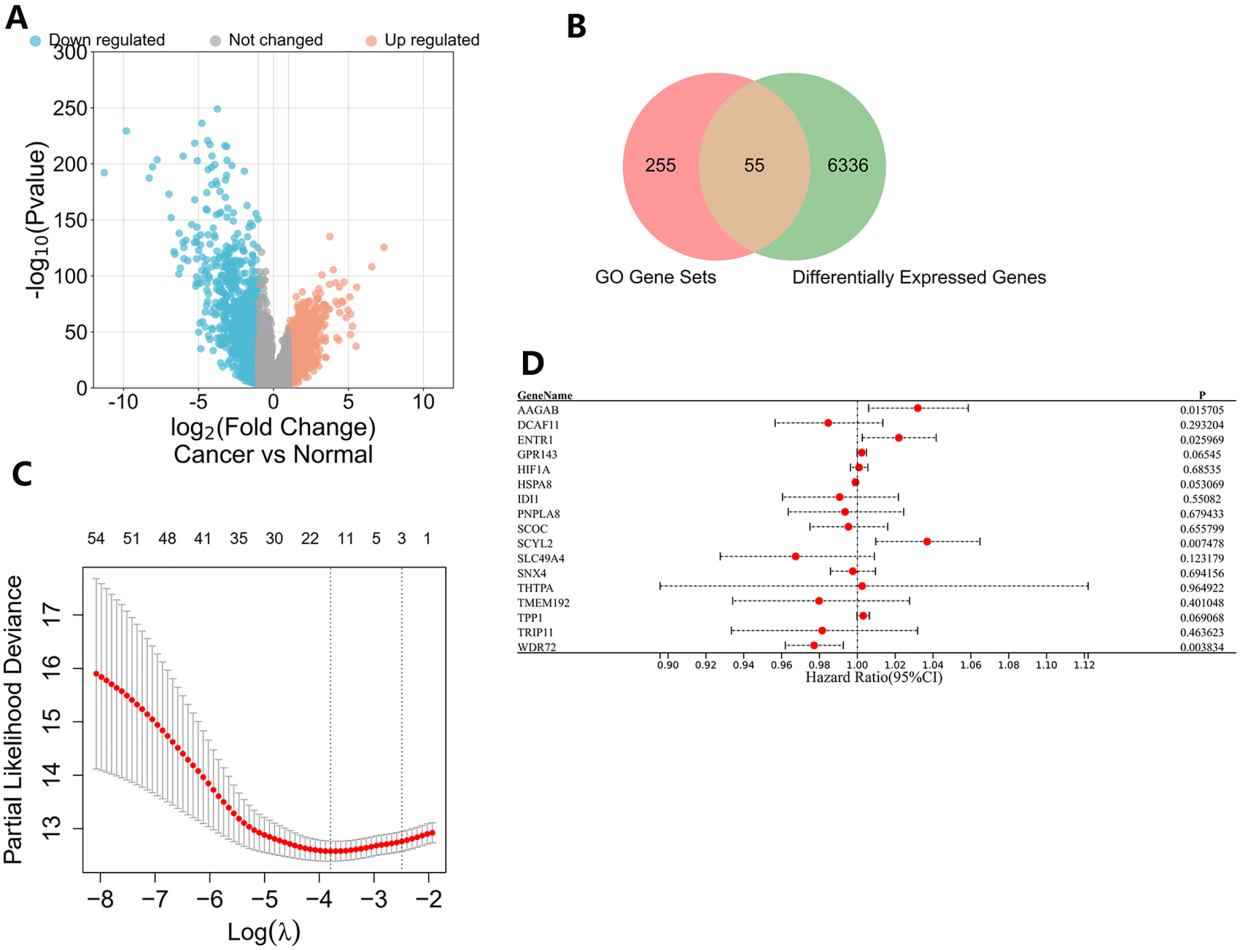
Screening of prognostic hub genes in the experimental group. **A** Differential gene expression analysis between cancer and normal samples. **B** Venn plot for the intersection of the 310 genes in the 20 enriched gene sets and DEGs between cancer and normal samples. **C** LASSO regression analysis to reduce variables. **D** Multivariate Cox regression analysis of 17 prognostic genes

To explore differentially expressed genes (DEGs) between cancer and normal samples, differential gene analysis in the experimental group was performed (Supplementary Table S10). As shown in Fig. 6A, 6391 DEGs were identified according to the criteria of |Log2FC|> 0.5 and P value < 0.05. Finally, we identified 55 research genes to construct a risk model from the intersection of 310 genes of the 20 enriched gene sets and DEGs (Fig. 6B, Supplementary Table S11).

### Construction of a risk model and identification of clinical relevance

To investigate the prognostic values of the 55 research genes and filter out crucial genes, the LASSO regression analysis was first performed to reduce variables based on the experimental group (Fig. 6C). 17 genes were identified to have vital prognostic values in 55 research genes (Supplementary Table S12). Subsequently, the influences of the 17 genes on the clinical outcomes of experimental group samples were evaluated by the multivariate Cox regression analysis (Fig. 6D). Finally, we obtained four hub genes including AAGAB, ENTR1, SCYL2, and WDR72 that had independent prognostic values to construct a risk model. The AAGAB and the ENTR1 expressions showed significant differences in the different T stages and different tumor stages (Fig. S3C, D). The WDR72 expressions were also found to be associated with the stage information in the experimental group (Fig. S3A). The risk scores of every sample in experimental and validation groups were calculated as follows:

$$\begin{aligned} {\text{risk score }} &= {\text{Expr}}\left( {{\text{AAGAB}}} \right) \times \left( {0.031} \right) \\ &\quad+ {\text{ Expr}}\left( {{\text{ENTR}}1} \right) \times \left( {0.022} \right) \\ &\quad+ {\text{ Expr}}\left( {{\text{SCYL}}2} \right) \times \left( {0.036} \right) \\&\quad + {\text{ Expr}}\left( {{\text{WDR}}72} \right) \times \left( { - 0.023} \right). \\ \end{aligned}$$

**Fig. 7:**
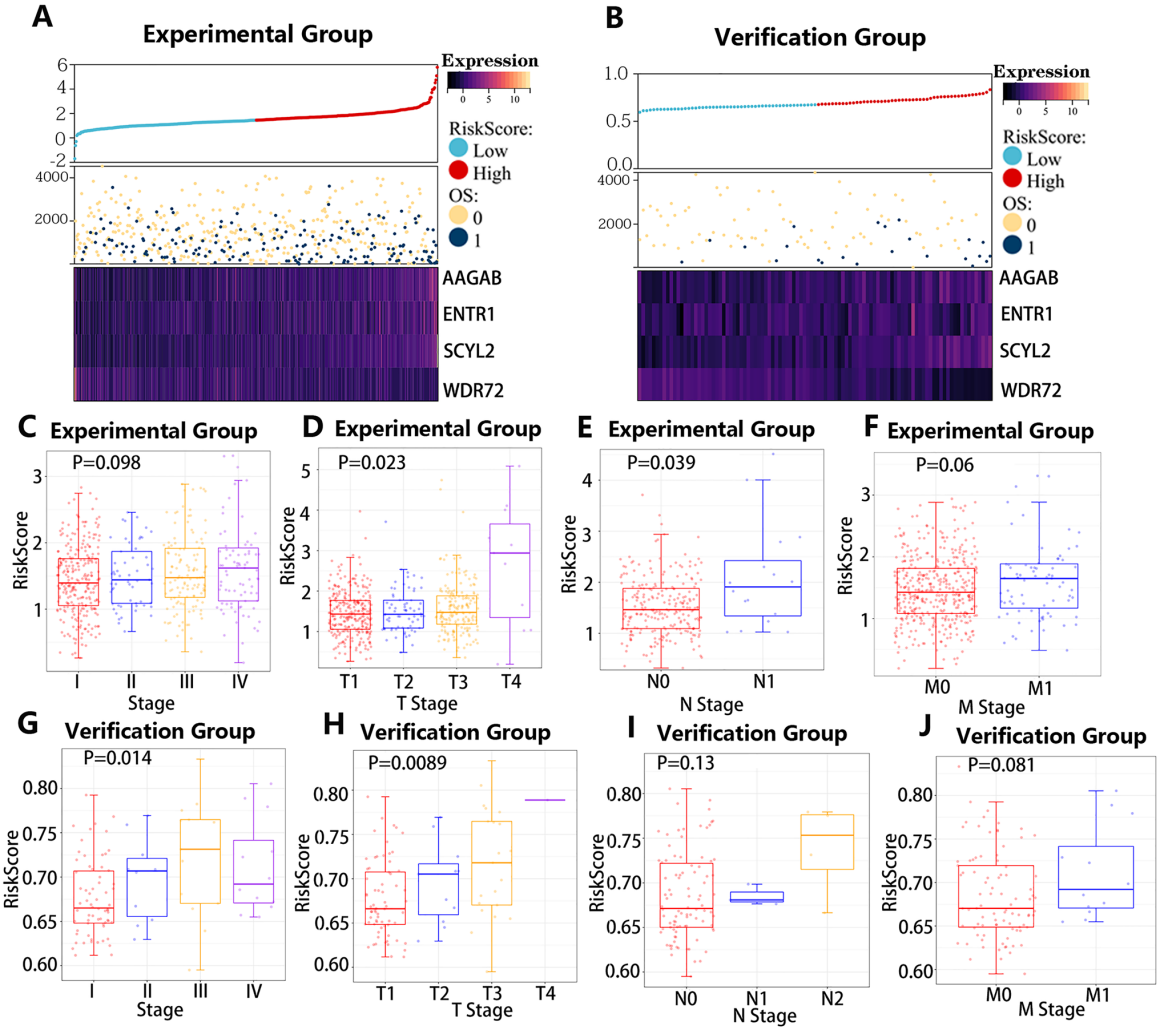
Clinical values of the risk score. **A** Distribution of the overall survival (OS) time and the expressions of four hub genes in the TCGA ccRCC cohort. **B** Distribution of the overall survival (OS) time and the expressions of four hub genes in the E-MTAB-1980 ccRCC cohort. **C–F** Associations between the risk score and some vital clinicopathological factors in TCGA ccRCC cohort. **G–J** Associations between the risk score and some vital clinicopathological factors in the E-MTAB-1980 cohort

To explore the impacts of the risk model on the clinical prognosis, all samples were divided into a low-risk group and a high-risk group according to the median cutoff of 1.45262 and 0.674556 in experimental and validation groups. The different distribution of the overall survival (OS) time and the expressions of four hub genes between different risk groups are shown in Fig. 7A, B. Risk scores were found to be closely associated with some vital clinicopathological factors in experimental (Fig. 7C–F) and validation (Fig. 7G–J) groups. In all, our risk model was significantly correlated with the prognosis of ccRCC.

### Construction of an individualized predictive model

**Fig. 8:**
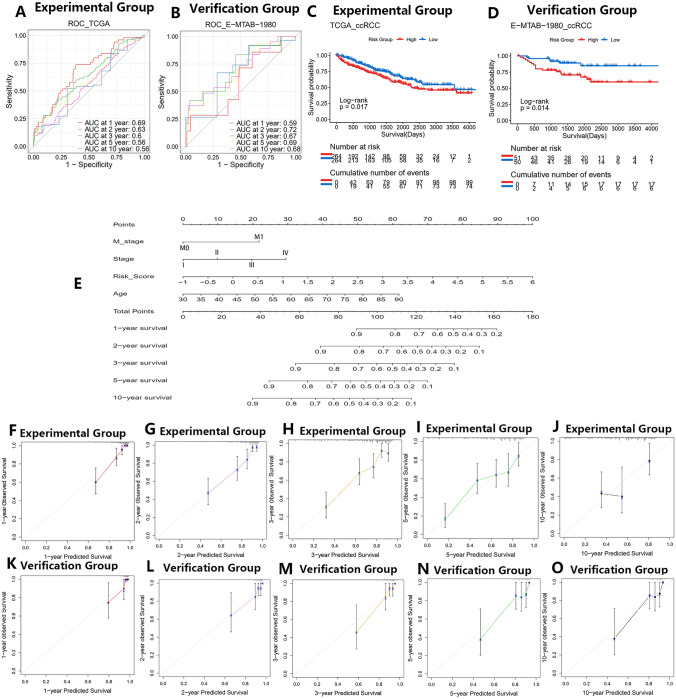
Evaluation of predictive values of the risk score and construction of an individualized predictive model. **A**, **B** ROC curves of the risk score in TCGA and E-MTAB-1980 ccRCC cohorts. **C**, **D** KM curves based on risk groups in TCGA and E-MTAB-1980 ccRCC cohorts. **E** Nomogram of the individualized Cox proportional hazard regression model. **F–J** Calibration curves of the individualized predictive model in TCGA ccRCC cohort. **K–O** Calibration curves of the individualized predictive model in E-MTAB-1980 ccRCC cohort

We drew ROC curves of the risk scores in experimental and validation groups to check the discrimination ability of the risk model. The AUC for 1, 2, 3, 5, and 10 years was 0.69, 0.63, 0.6, 0.56, and 0.56, respectively, in the experimental group (Fig. 8A). In the E-MTAB-1980 cohort, the AUC for 1, 2, 3, 5, and 10 years was 0.59, 0.72, 0.67, 0.69, and 0.68, respectively (Fig. 8B). Meanwhile, KM curves drawn according to the risk groups showed that overall survival rates of the high-risk subtype were markedly lower than those in the low-risk subtype (Fig. 8C, D). In all, the above results indicated that the risk model had some predictive value for the survival outcomes in ccRCC.

To construct a more accurate individualized predictive model, some other possible risk factors (age, gender, race, M stage, N stage, T stage, stage, and prior malignancy history) were incorporated into this study with the risk score. We performed the stepwise multivariate Cox regression analysis and then obtained four predictive factors (M stage, N stage, stage, risk score, and age) according to the minimum AIC index 861.26 (Table S13). To decrease predictive errors, the N stage factor with *P* value = 0.1287 was excluded. Finally, a multivariate Cox proportional hazard regression model including M stage, stage, risk score, and age based on the experimental group was constructed and then quantified into a nomogram plot (Fig. 8E, Table S14).

C-index was usually calculated to evaluate the discrimination ability of the Cox model. In this study, C-index for the whole mode was 0.7620 in the experimental group. For the external validation group, the C-index was 0.7920. In addition, calibration curves showed good predictive accuracy in both experimental (Fig. 8F–J) and validation (Fig. 8K–O) groups. All the above results indicated good discrimination ability and accuracy of the predictive model.

### GSEA between different risk groups

GSEA was performed between two risk groups by GSEA 4.3.2 analysis software according to GO and KEGG official candidate gene sets in this study. We selected significantly enriched gene sets according to the criterion of NOM *P*-val < 0.05 and FDR < 0.25 (Data. S1). According to the GSEA results, some pathways that might correlate with AAMR were found to be significantly enriched in the high-risk group and Fig. S4 showed 16 most significant ones of them. Our results uncovered that TMEM251-related AAMR pathways might be potential mechanisms for the risk model to influence ccRCC prognosis.

### Associations between the risk score and TIME

**Fig. 9:**
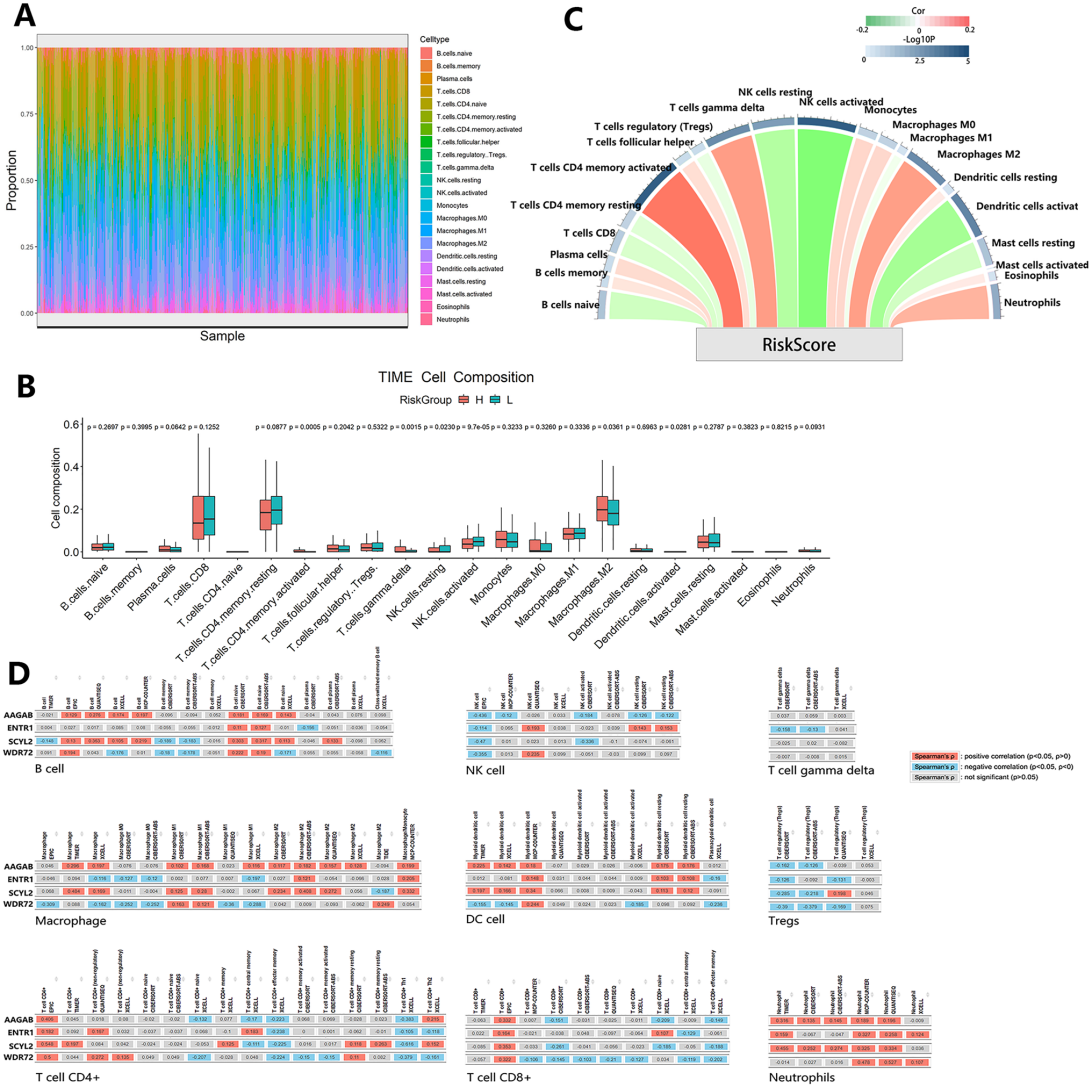
Immune infiltration analysis. **A** Immune cell infiltration proportion of every patient. **B** Differences of 22 immune cell infiltration levels between risk groups. **C** Spearman correlation analysis between the risk score and immune cell infiltration levels. **D** Spearman correlation analysis between four hub genes and immune cell infiltration levels based on the TIMER database

Immune cell infiltration analysis was usually used to explore the composition characteristics of TIME. Immune cell infiltration ratios of every patient are shown in Fig. 9A. In this study, infiltration scores of 22 common immune cells were calculated by the CIBERSORT algorithm (Table S15) and were compared between different risk groups to reveal the impacts of the risk score model on TIME (Fig. 9B). Infiltration levels of activated memory CD4 + T cells, gamma delta T cells, and M2 macrophages were higher in the high-risk group. But for resting and activated NK cells, infiltration levels were lower in the high-risk group than that in the low-risk group. Meanwhile, the heatmap (Fig. S5) also showed a different distribution of immune cell infiltration proportion between different risk groups. To better describe specific connections between the risk score model and TIME, Spearman correlation analysis was performed. The risk score was found to be significantly positively correlated with memory CD4 + T cells, gamma delta T cells, and M2 macrophages but negatively correlated with resting NK cells, activated NK cells, and activated dendritic cells (Fig. 9C). In conclusion, our analysis suggested that the risk score model could influence the TIME of ccRCC mainly by changing T cells, macrophages, and NK cell infiltration levels.

Moreover, to explore the impacts of four hub genes on TIME and immunotherapy responsiveness, Spearman correlation analysis results between these genes and nine common immune cells (B cell, dendritic cell, macrophage, neutrophil, NK cell, CD4 + T cell, CD8 + T cell, gamma delta T cell, and Tregs) infiltration levels were obtained from the TIMER database (Fig. 9D). AAGAB, as a risk gene, was positively correlated with CD4 + T cell, Th2 CD4 + T cell, B cell, macrophage, and dendritic cell infiltration levels, but it was negatively correlated with memory CD4 + T cell and Tregs infiltration levels. It was worth mentioning that AAGAB was also negatively correlated with NK cell infiltration levels no matter which calculation methods on the TIMER database were used. For ENTR1, it was positively correlated with CD4 + T cell, NK cell, M2 macrophage, and dendritic cell infiltration levels, but it was negatively correlated with M0 macrophage, M1 macrophage, Tregs, and gamma delta T cells infiltration levels. The impacts of AAGAB and ENTR1 on the TIME were similar except for the NK cell. SCYL2, as another risk gene, showed positive correlations with CD4 + T cell, memory CD4 + T cell, macrophage, and dendritic cell infiltration levels. Meanwhile, SCYL2 showed a more strongly negative correlation with the NK cell than the other genes. Finally, WDR72 was found to be positively correlated with CD4 + T cell infiltration levels by the EPIC method, but it was negatively correlated with naïve and memory CD4 + T cells by other methods. WDR72 also showed negative correlations with Tregs and CD8 + T cell infiltration levels. Moreover, we found that all hub genes were positively correlated with neutrophil infiltration levels. In conclusion, the four hub genes were demonstrated to possess possible vital functions in regulating the TIME of ccRCC and predicting the immunotherapy responsiveness.

### Tumor mutation burden (TMB) analysis

**Fig. 10:**
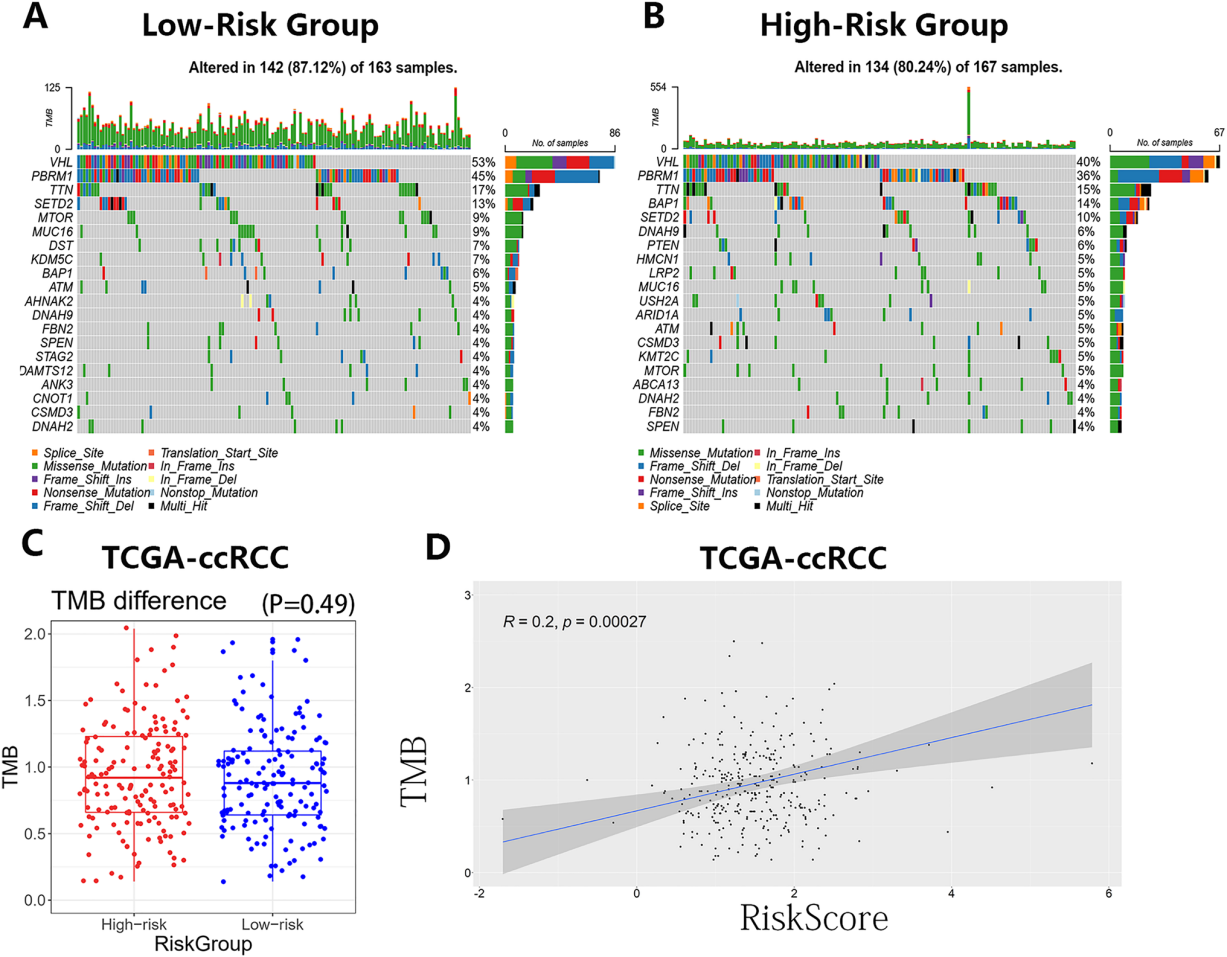
Tumor mutation burden (TMB) analysis. **A** Gene mutation analysis in the low-risk group. **B** Gene mutation analysis in the high-risk group. **C** Associations between the TMB score and the risk group. **D** Pearson correlation analysis between the risk score and the TMB score

A total of 330 samples were divided into the low-risk and the high-risk group according to the median cutoff of 1.460753. Gene mutation information of all samples was shown in Fig. S6A. Figure S6B, C showed different gene mutation patterns between the risk groups. As far as the top 20 mutation genes were concerned, the two risk groups were almost completely different (Fig. 10A, B). The TMB score of every sample was calculated by R software (Table S16). In addition, TMB scores were higher in the high-risk group, but the difference was not statistically significant (Fig. 10C). However, through the Pearson correlation analysis, we found that the risk score was positively correlated with the TMB score (Fig. 10D).

### Predictive value of the risk score for the immunotherapy responsiveness

**Fig. 11:**
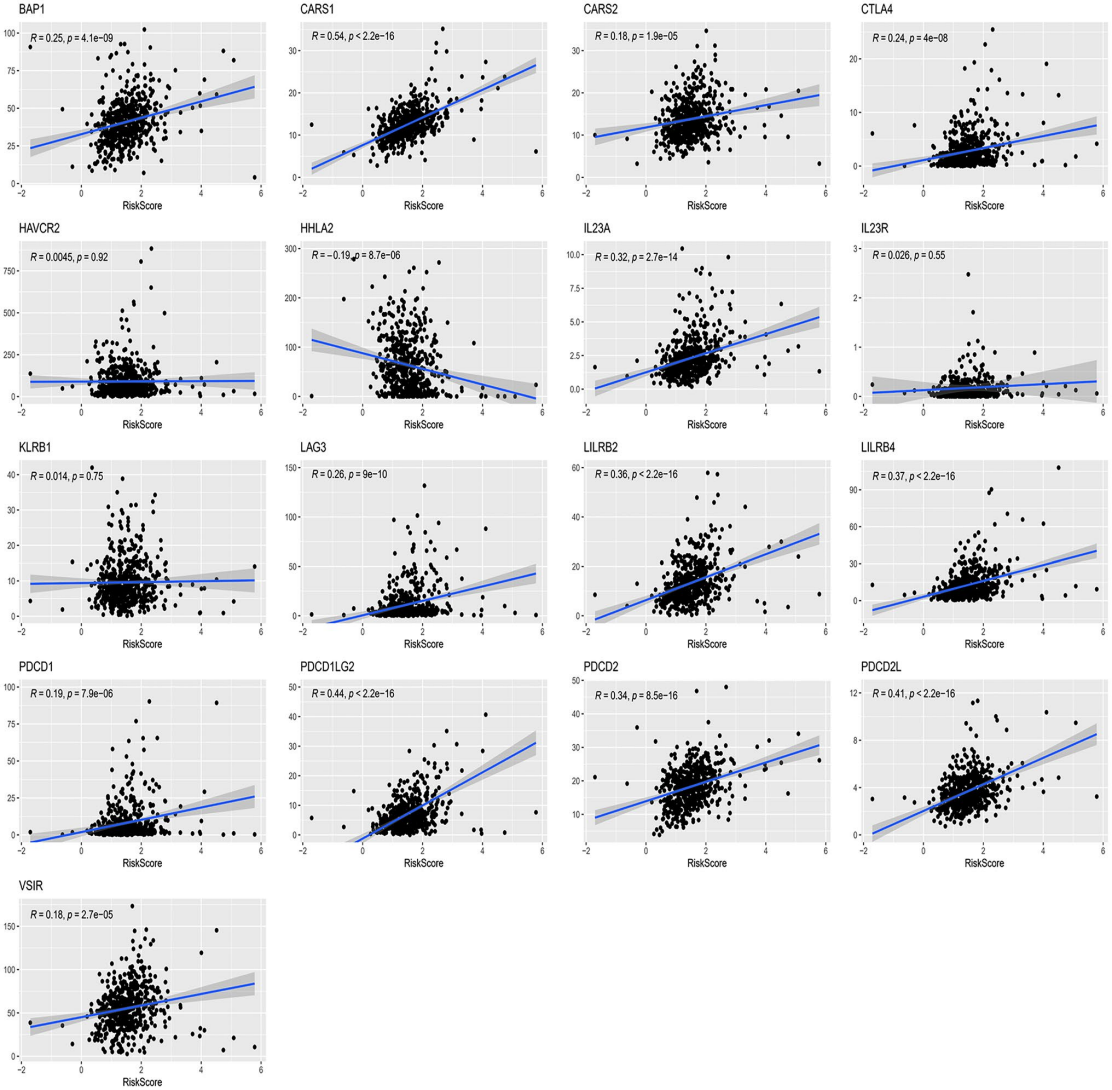
Pearson correlation analysis between the risk score and 17 common immune checkpoint genes

**Fig. 12:**
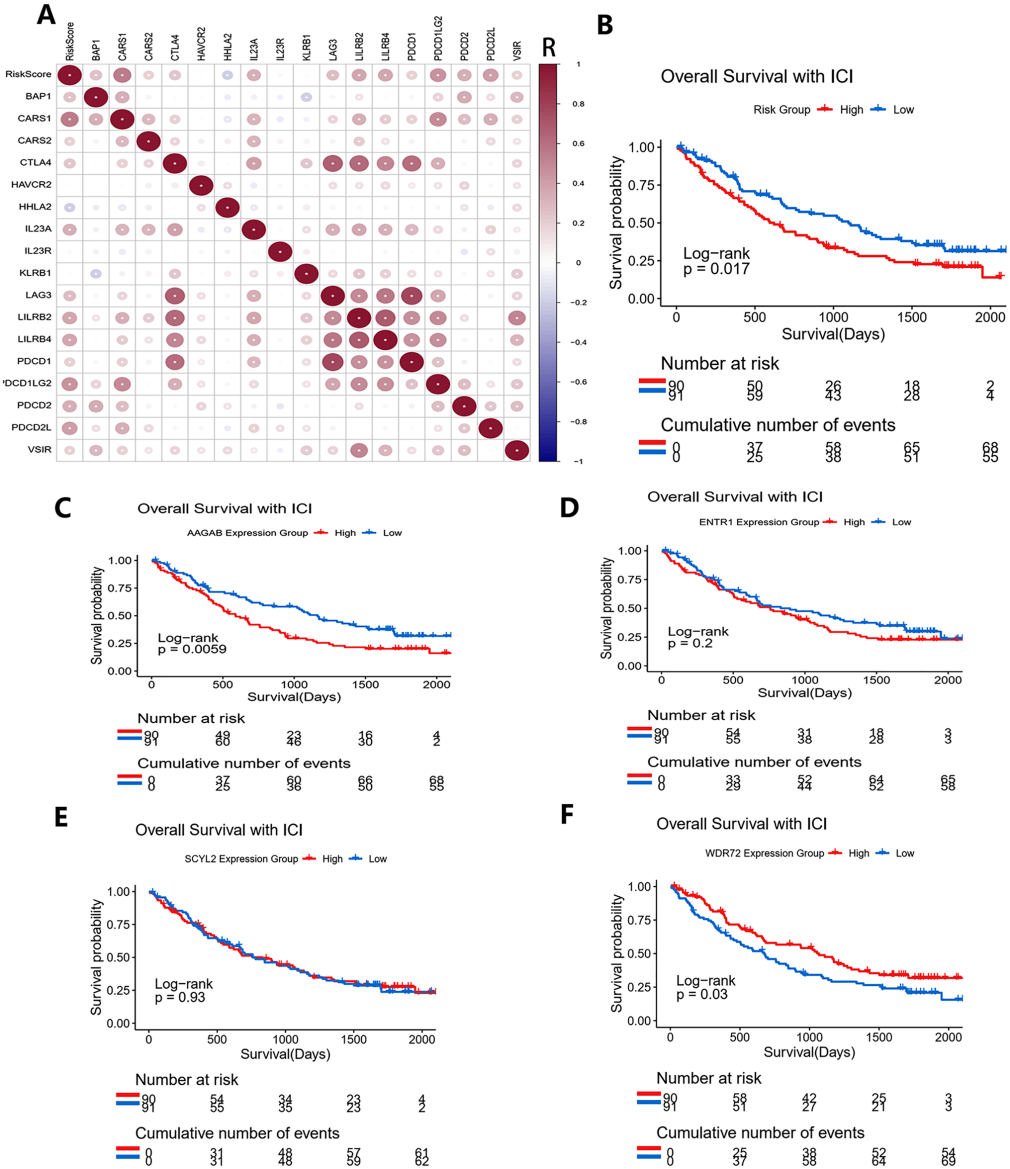
Revealing predictive values of the risk score for immunotherapy responsiveness based on CM-009, CM-010, and CM-025 immunotherapy cohorts. **A** Pearson correlation analysis among 17 common immune checkpoint genes. **B** KM curves based on different risk groups. **C–F** KM curves based on different expression groups of four hub genes

Expression levels of 17 common immune checkpoint genes (BAP1, CARS1, CARS2, CTLA4, HAVCR2, HHLA2, IL23A, IL23R, KLRB1, LAG3, LILRB2, LILRB4, PDCD1, PDCD1LG2, PDCD2, PDCD2L, and VSIR) were calculated in the experimental group. Pearson correlation coefficients and corresponding P values were obtained by batch Pearson correlation analysis. The strengths of the linear correlations between the risk score and the immune checkpoint genes are shown in Fig. 11. Sixteen immune checkpoint expressions were positively correlated with the risk score, and only HHLA2 was negatively correlated. CARS1, PDCD1LG2, PDCD2L, LILRB4, and LILRB2 showed the strongest correlation with the risk score in this study. Positive correlations among these immune checkpoint genes are shown in Fig. 12A. Finally, the impacts of the risk score and four hub genes on ICI (Nivolumab) therapy responsiveness were evaluated based on the immunotherapy cohort. Survival analysis showed that the higher risk score and the higher AAGAB expression correlated with the lower overall survival rates for patients with Nivolumab therapy (Fig. 12B, C), but the higher WDR72 expression was associated with higher overall survival rates (Fig. 12F). ENTR1 and SCYL2 expressions had no significant impacts on the overall survival rates of the patients with Nivolumab therapy. The above results indicated potential predictive values of the risk score, AAGAB, and WDR72 for immunotherapy responsiveness in ccRCC.

### Single-cell sequencing analysis

**Fig. 13:**
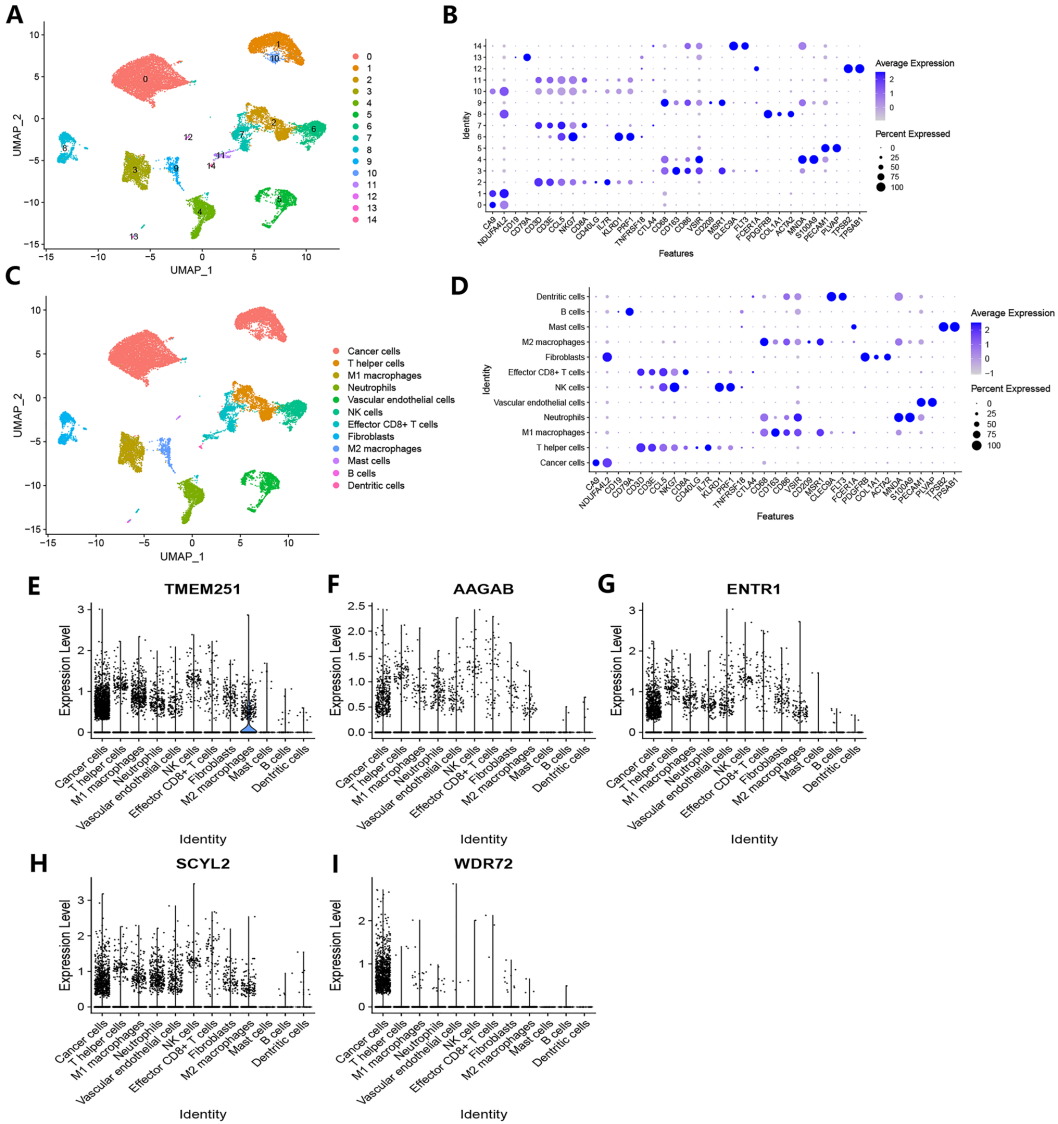
Single-cell sequencing analysis. **A** UMAP plot of initial cell cluster result. **B** Expressions of cell marker genes in different cell clusters. **C** UMAP plot of annotated cell clusters. **D** Expressions of cell marker genes in annotated cell clusters. E-I Expressions of TMEM251 and 4 hub genes on specific cell clusters

After initial screening, 19,453 cells were finally included in subsequent analysis. These cells were clustered into 15 groups (Fig. 13A). Based on classical cell markers (Table S17 and Fig. 13B) from CellMarker 2.0 database, all cell groups were annotated (Fig. 13C, D). The TMEM251, AAGAB, ENTR1, SCYL2, and WDR72 were all found to be expressed mainly in cancer cells (Fig. 13E–I). It is worth mentioning that these genes except for WDR72 were also expressed in immune cells and fibroblasts.

### Protein-level verification of the four hub prognostic genes

**Fig. 14:**
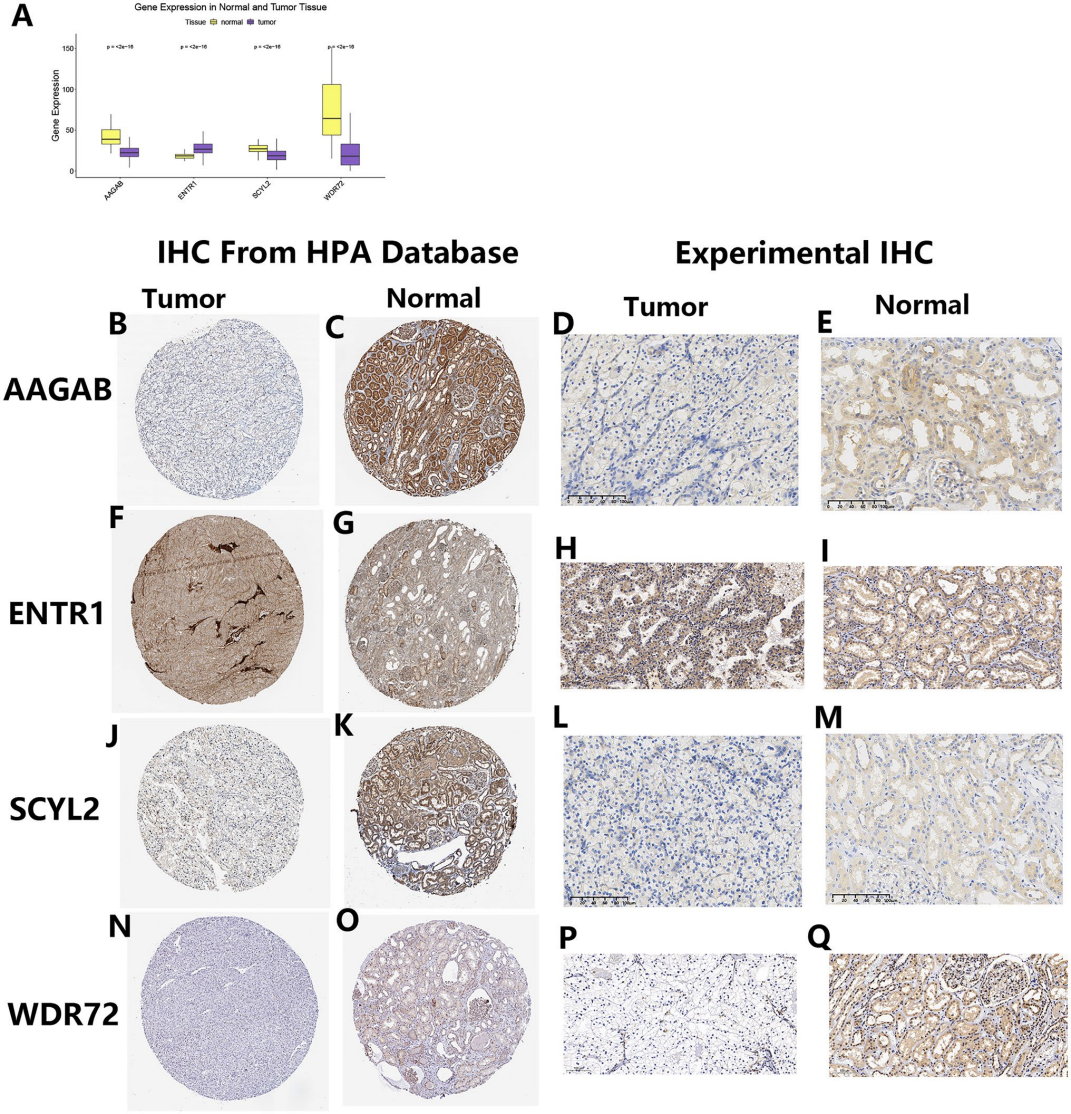
Protein-level verification of the four hub prognostic genes. **A** Differential mRNA expression analysis of 4 hub genes. **B**–**E** Immunohistochemical staining of AAGAB. **F**–**I** Immunohistochemical staining of ENTR1. **J**–**M** Immunohistochemical staining of SCYL2. **N**–*Q* Immunohistochemical staining of WDR72

Consistent with the differential mRNA expression analysis result, the corresponding protein expression levels of ENTR1 were found to be higher in tumor samples (Fig. 14A, F–I). However, as risk genes for poor prognosis, the AAGAB and the SCYL2 expressions were found to be decreased in the tumor tissues at both mRNA and protein levels (Fig. 14A, B–E, J–M). We also thought this was correlated with the dual role of autophagy in tumor development and possible reasons were discussed in the discussion section. As expected, the WDR72 was found to be expressed at a lower level in tumor sample as a protective gene (Fig. 14A, N–Q).

## Discussion

The latest studies have found that LYSET, as a transmembrane protein encoded by TMEM251, is a key factor for keeping normal components and protein degradation functions of the lysosomal. In the nutrient-deficient TME, LYSET plays an essential role in cancer cells’ AAMR to enhance extra protein degradation for amino acid supplements. However, specific biological functions and pathways of the LYSET in ccRCC are still unclear (Richards et al. 2022; Zhang et al. 2022a; Pechincha et al. 2022). In this study, we first analyzed the clinical correlation of the TMEM251 expression and found a dual role of the TMEM251 expression in ccRCC development. Possible explanations of this conclusion are as follows: The LYSET encoded by TMEM251 is a key regulator for normal lysosomal components and functions, so the TMEM251 expression might reflect numbers and functions of the lysosome. The lysosome-mediated autophagy is widely thought to play a dual role in tumor development. In early tumorigenesis, autophagy, as a survival pathway of cells to eliminate damaged proteins and organelles for quality control, prevents tumor initiation and development. Once the tumors were in the advanced stages, autophagy, as a recycling system for nutrient supplements to meet excessive nutrient and energy demands of tumor cells, contributes to the survival and growth of tumors (Li et al. 2020; White 2012). Therefore, the TMEM251 expression showed a dual role in ccRCC development mainly because of the TMEM251-related lysosome-mediated autophagy. The analysis results of GSVA are also similar. Subsequently, we analyzed the biological functions and pathways of LYSET in ccRCC using GO and KEGG methods. GO analyses showed the most enriched biological processes were vesicle-mediated transport, intracellular protein transport, and protein transport. It is widely recognized that vesicle-mediated transport is included in lysosome biosynthesis and utilization of extracellular protein (Tejeda-Munoz et al. 2019; Ford et al. 2021). Meanwhile, the TCA cycle, proteasome, and metabolic pathways were three significantly enriched gene sets in KEGG, which indicated that the LYSET was highly correlated with cell metabolism pathways. Although the point that glycolysis is the main energy metabolism pathway instead of oxidative phosphorylation or TCA cycle for cancer cells has been widely accepted, α-ketoglutarate, as the downstream metabolites of glutamine can be still transported into mitochondrion for supplementing the TCA cycle (Still and Yuneva 2017; Koppula et al. 2018; Liberti and Locasale 2016). It is worth noting that some gene sets related to the mitochondrion and TCA cycle were enriched in our study, which meant that TMEM251 expression possibly correlated with enhanced glutamine metabolism. In addition, we found that 20 gene sets that might be associated with AAMR were also enriched in GO and KEGG results. These gene sets mainly referred to endocytosis, lysosome-mediated protein degradation, autophagy, and proteasome-mediated protein degradation. Several studies have proven that lysosome-mediated autophagy and extracellular protein degradation are important ways of cancer cells' amino acid supplement in nutrient-deficient conditions (Martinez-Reyes and Chandel 2021). For example, in breast cancer, the rapid proliferation of tumors caused an amino acid deficiency in TME, but tumor cells could still obtain enough amino acid by enhancing protein endocytosis and lysosomal degradation (Corchado-Cobos et al. 2022). Enhanced endocytosis and lysosome-mediated autophagy for adaption to poor amino acid supplements have been also observed in pancreatic cancer (Kamphorst et al. 2015). In addition, our results showed that proteasome-related biological process was also active when TMEM251 was highly expressed. Although we have not found enough experiments to show amino acid supplement functions of proteasome-mediated degradation in cancer cells, the ubiquitin–proteasome system is responsible for approximately 80–90% cellular proteolysis and maintains amino acid supplement synergizing with the lysosome system for normal cells in nutrient-deficient conditions (Rousseau and Bertolotti 2018; Suraweera et al. 2012; Kwon and Ciechanover 2017; Vabulas and Hartl 2005). Therefore, we infer proteasome-mediated degradation is also a possible pathway for supplement according to the enriched results. Bioinformatics analysis results in this study indicated that LYSET might be associated with normal components and functions of the lysosome and guarantee the protein degradation process for amino acid supplements in ccRCC with nutrient-deficient TME. To our best knowledge, this is the first study to analyze biological functions and pathways of LYSET in ccRCC by bioinformatics methods.

To reveal the prognostic values of LYSET and related biological pathways in ccRCC, GSVA and unsupervised cluster analysis were used. In the initial analysis, we found significant TMEM251 expression differences in different T stages and different tumor stages. GSVA results also showed higher enrichment scores of the 20 enriched gene sets were all significantly correlated with the later T stages. It indicated that TMEM251 might influence the prognosis by affecting tumor volume. Cancer cells uptake more glucose for glycolysis instead of oxidative phosphorylation even in oxygen-abundant conditions to meet high energy requirements. This phenomenon is called Warburg effect (Liberti and Locasale 2016). However, the latest study found that M2 macrophage takes up and consumes the largest amounts of glucose in TME instead of tumor cells (Shi et al. 2022). Thus, AAMR plays a more and more important role in cancer metabolism. For example, glutamine addiction promotes cancer cells' defense against oxidative stress. And the downstream metabolites of tryptophan boost cancer growth and help evade the natural immune system in ccRCC (Chakraborty et al. 2021). Moreover, arginine deprivation has been also proven to suppress tumor growth in ccRCC (Yoon et al. 2007). In our study, different survival rates among clusters in cluster analysis showed the prognostic values of the LYSET and related pathways in ccRCC. As far as the prognostic values of the LYSET in ccRCC are concerned, we haven't found similar bioinformatics research.

CcRCC characterized by abundant altered metabolism pathways is common cancer with a poor prognosis in the advanced stages, but it has pretty good clinical outcomes in the early stages. Meanwhile, some patients with advanced ccRCC can’t benefit from immunotherapy (Campbell et al. 2017). Therefore, an accurate predictive tool for the prognosis and immunotherapy responsiveness is urgently needed for clinical choice. Existing models for ccRCC prognosis such as Memorial Sloan-Kettering Cancer Center (MSKCC), Cleveland Clinic Foundation (CCF), the French, and International Kidney Cancer Working Group (IKCWG) models are mostly based on clinical information and laboratory test results. In this study, we constructed a risk score model based on LYSET-related biological processes and pathways genes. Four crucial genes AAGAB, ENTR1, SCYL2, and WDR72 were identified to calculate the risk score by LASSO and COX analysis. Several studies have shown that these genes can influence the development of some cancers. Sharma S et al. confirmed that in ENTR1-depleted cells, transporting to lysosomes was significantly impaired and ENTR1 is a negative regulator of Fas cell surface levels and Fas apoptotic signals (Sharma et al. 2019). Neznanov N et al. confirmed that ENTR1 is a key regulator of TNFR expression levels and TNF-induced signaling pathways (Neznanov et al. 2005). Hagemann, N et al. found that ENTR1 was highly expressed in colon cancer and significantly influenced cancer development (Hagemann et al. 2013). Liu, H et al. confirmed that EPRS bound with SCYL2 to enhance activation of the WNT/GSK-3β/β-catenin signaling pathway and accumulation of β-catenin in nuclear, leading to gastric cancer cell proliferation and growth (Liu et al. 2021). Mares, J et al. confirmed that WDR72 is correlated with the recurrence of bladder cancer (Mares et al. 2013). ROC curves and KM curves in this study both indicated potential prognostic values of the risk score model. Finally, we furtherly constructed an individualized predictive model. C-index and calibration curves showed good discrimination ability and predictive accuracy of the model. Similarly, on the foundation of a group of vital genes in a specific biological process, there have been many models related to metabolism gene signatures for prognosis prediction in ccRCC (Xie et al. 2022; Liu et al. 2020; Chen et al. 2021; Guo et al. 2021). Although these models were constructed based on cancer metabolism genes and were validated in different ways, they all referred to the whole metabolism pattern of ccRCC and did not discuss the prognostic values of specific amino acid metabolism genes. Moreover, these studies mainly paid attention to normal metabolism pathway genes and didn't focus on abnormal metabolism in ccRCC. Although Zhang, Q et al. have constructed a model based on abnormal metabolism genes in ccRCC, they still didn’t focus on specific AAMR (Zhang et al. 2022b). In addition, Zhang, F et al. and Cheng, X et al. have established two risk models based on amino acid metabolism gene sets recently, but they didn't consider transformed amino acid metabolism pathways in ccRCC (Cheng et al. 2022; Zhang et al. 2022c). However, in this study, we focused on the LYSET-related AAMR process. These metabolism processes are usually active in cancer cells but are not very common in normal cells. Meanwhile, prognostic analysis of LYSET and construction of the risk model based on LYSET-related gene signatures also help to uncover the impacts of LYSET on the progression and development of ccRCC.

To reveal the underlying mechanisms of the risk model on prognosis in ccRCC, immune infiltration analysis in TIME was performed. TIME is the immune infiltrative microenvironment of a tumor, which consists of a large number of immune cells within and around the tumor (Binnewies et al. 2018). Tumor cells closely interact with TIME through different metabolic pathways. Metabolism reprogramming can cause increased requirements of energy in tumors and relative shortages of nutrients in TIME, finally leading to immune cell function inhibition. Meanwhile, tumor cells can influence the functions of surrounding immune cells by releasing metabolites such as lactic acid, PGE2, and arginine (Xia et al. 2021). A previous study confirmed that exogenous arginine supplementary in TIME could change glucose metabolism pathways of T cells and significantly improve the anti-tumor ability of CD4 + , CD8 + , and memory T cells (Geiger et al. 2016). Arginine supplements in TIME also can enhance the cytotoxic functions of T cells and NK cells, and they improve ICI therapy responsiveness in osteosarcoma (He et al. 2017). In addition, a previous study indicated that the activation of T cells is sensitive to tryptophan concentrations, but large uptake of tumor cells could cause tryptophan shortage and lead to apoptosis of T cells (Cronin et al. 2018). Tryptophan shortage also promoted tumor immune escape by inducing Treg infiltration, downregulation of CD8 + T cell numbers, and upregulation of inhibitory receptors ILT3 and ILT4 on dendritic cells (Opitz et al. 2020). Therefore, metabolism patterns especially amino acid metabolism of tumor cells can significantly influence immune cell status in TIME. In this study, we calculated the infiltration levels of 22 common immune cells in ccRCC based on the TCGA cohort. Memory CD4 + T cell, gamma delta T cell, and M2 macrophage infiltration were found to be positively correlated with the risk score, but NK cell and dendritic cell infiltration were negatively correlated with the risk score. Xu, L et al. confirmed that a higher proportion of M2 macrophage infiltration in ccRCC TIME is associated with a worse prognosis (Xu et al. 2014). Yet, a higher proportion of NK cells was found to be associated with better prognosis in ccRCC (Remark et al. 2013). For T cells, as the major immune cells in ccRCC (Chevrier et al. 2017), their low-level infiltration might correlate with a worse prognosis. Our analysis results indicated that risk score could influence the prognosis of ccRCC by mainly changing T cell, M2 macrophage, and NK cell infiltration levels in TIME. A possible potential mechanism was that the high-risk score meant enhanced LYSET-related AAMR in tumor cells that was usually observed in amino acid-deficient conditions (Pechincha et al. 2022). As shown in previous studies, deficiency of crucial amino acids in TIME can influence normal immune infiltration and immune cell functions (Xia et al. 2021; Geiger et al. 2016; He et al. 2017; Cronin et al. 2018; Opitz et al. 2020). In addition, as shown in the previous part, expression levels of four key risk genes were also found to be closely correlated with immune cell infiltration levels. Previous studies have shown that higher NK cell infiltration proportion was associated with better prognosis in ccRCC (Remark et al. 2013). CD8 + T cell and neutrophil infiltration were also thought to be connected with the anti-tumor immune effect (Angell et al. 2020; Wang et al. 2018). It is particularly noteworthy that AAGAB was negatively correlated with NK cell infiltration and WDR72 significantly negatively correlated with CD8 + T cell infiltration in this study. Four hub genes were all found to be positively correlated with neutrophil infiltration level.

Immune cell status in TIME is thought to be an important predictor of immunotherapy responsiveness (Sharma et al. 2017). The immunosuppressive tumor microenvironment remains the biggest obstacle to immunotherapy efficacy (Lequeux et al. 2021). Previous results in our study have indicated that the risk model based on LYSET-related AAMR genes is closely correlated with TIME in ccRCC. Thus, we explored connections between the risk model and immunotherapy responsiveness. Based on the TCGA cohort, only except for HHLA2, the other 16 common immune checkpoint expressions were revealed to be positively correlated with TMEM251 expression, which indicated possible ICI therapy benefit in patients with high TMEM251 expression. Meanwhile, we found that the risk score was positively correlated with the tumor mutation burden score. Moreover, through survival analysis in the immunotherapy cohort, we found that higher risk scores and AAGAB expressions correlated with lower survival rates for patients with Nivolumab therapy but higher WDR72 expression was associated with higher survival rates. It indicated that our risk model might have some predictive value for ICI therapy in ccRCC based on CM-009, CM-010, and CM-025 cohorts, but these results need to be validated by more experiments.

Through single-cell sequencing analysis, we found the TMEM251 and the four hub prognostic genes were mainly expressed in the cancer cell. Interestingly, these genes were also found to be expressed in some immune cells. It indicated possible targets similar to the tumor cells in the immune cells of ccRCC. The results need more experimental verification. Finally, the IHC was performed to validate the expressions of the four hub prognostic genes. In this study, it is worth mentioning that the AAGAB and SCYL2 expressions were lower in the cancer samples, but there were found to be risk factors through the Cox analysis. Possible reasons are as follows: AAGAB and SCYL2 identified for the risk model construction were derived from the gene sets associated with lysosome-mediated autophagy. In early tumorigenesis, autophagy, as a survival pathway for cellular components quality control, prevents tumor initiation. Once the tumors were established and in advanced stages, autophagy, as a recycling system for nutrient supplements, contributes to the survival and growth of the established and advanced tumors (Li et al. 2020; White 2012). Therefore, the AAGAB and the SCYL2 might inhibit the tumorigenesis of ccRCC, while they became risk genes for poor prognosis when tumors were established.

## Conclusions

In this study, we uncovered partial biological functions and pathways of LYSET (TMEM251) in ccRCC by bioinformatics analysis. Meanwhile, we revealed prognostic values of LYSET-related biological pathways and established related risk and predictive models. Finally, we furtherly explored how risk score affected prognosis through TIME in ccRCC and evaluated its predictive values for immunotherapy responsiveness. To our best knowledge, this is the first study that focuses on LYSET in ccRCC by bioinformatics analysis and there is no such similar model before. Our results and models can help understand LYSET more deeply in ccRCC and are useful for clinical choice. But there are still some limitations to this study. Although external validation and experiments were included in our study, our results and models were mainly based on open databases and enough pieces of experimental evidence were still deficient. Therefore, more experimental results are required to support the conclusions of this study in the future.

## Supplementary Information

Below is the link to the electronic supplementary material.

Supplementary file1 (ZIP 557 KB)

Supplementary file2 (ZIP 28064 KB)

Supplementary file3 (DOCX 20 KB)

Supplementary file4 (ZIP 3199 KB)

## Data Availability

The datasets for this study can be found in TCGA cohort (https://portal.gdc.cancer.gov) and the ArrayExpress cohort (https://www.ebi.ac.uk/biostudies/arrayexpress).

## Abbreviations

AAMR: Amino acid metabolism reprogramming
ccRCC: Clear cell renal cell carcinoma
LYSET: Lysosomal enzyme trafficking factor
TCGA: The Cancer Genome Atlas
TME: Tumor microenvironment
TIME: Tumor immune microenvironment
GO: Gene Ontology
KEGG: Kyoto Encyclopedia of Genes and Genomes
PPI: Protein–Protein Interaction Networks
GSVA: Gene Set Variation Analysis
DEGs: Differentially expressed genes
GSEA: Gene Set Enrichment Analysis
LASSO: Least absolute shrinkage and selection operator
KM: Kaplan–Meier
ICI: Immune checkpoint inhibitor
TMB: Tumor mutation burden
TNF: Tumor necrosis factor
TNFR: Tumor necrosis factor receptor
PCA: Principal component analysis
UMAP: Uniform manifold approximation and projection
