# Supplementary figures and images for: Functional enrichment analysis of LYSET and identification of related hub gene signatures as novel biomarkers to predict prognosis and immune infiltration status of clear cell renal cell carcinoma
Source: J Cancer Res Clin Oncol. 2023 Sep 23;149(18):16905–29. doi: 10.1007/s00432-023-05280-2 (PMC10645642; doi:10.1007/s00432-023-05280-2)

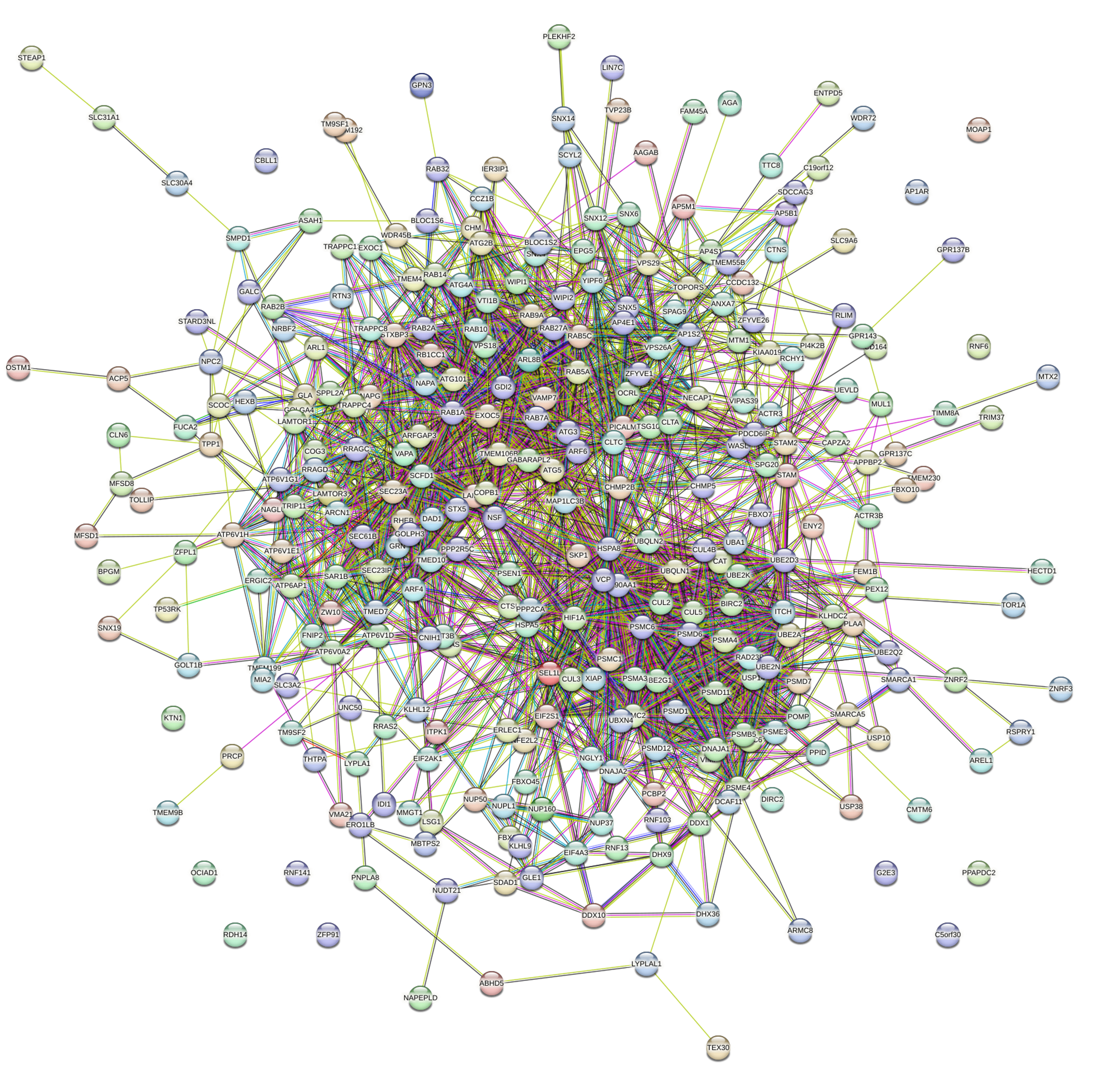

Supplement: Supplementary file 2 — Supplementary file2 (ZIP 28064 KB) [file 432_2023_5280_MOESM2_ESM.zip › Supplementary Figures/Supplementary Figure 1. PPI networks of 310 genes from 20 enriched gene sets.tif]

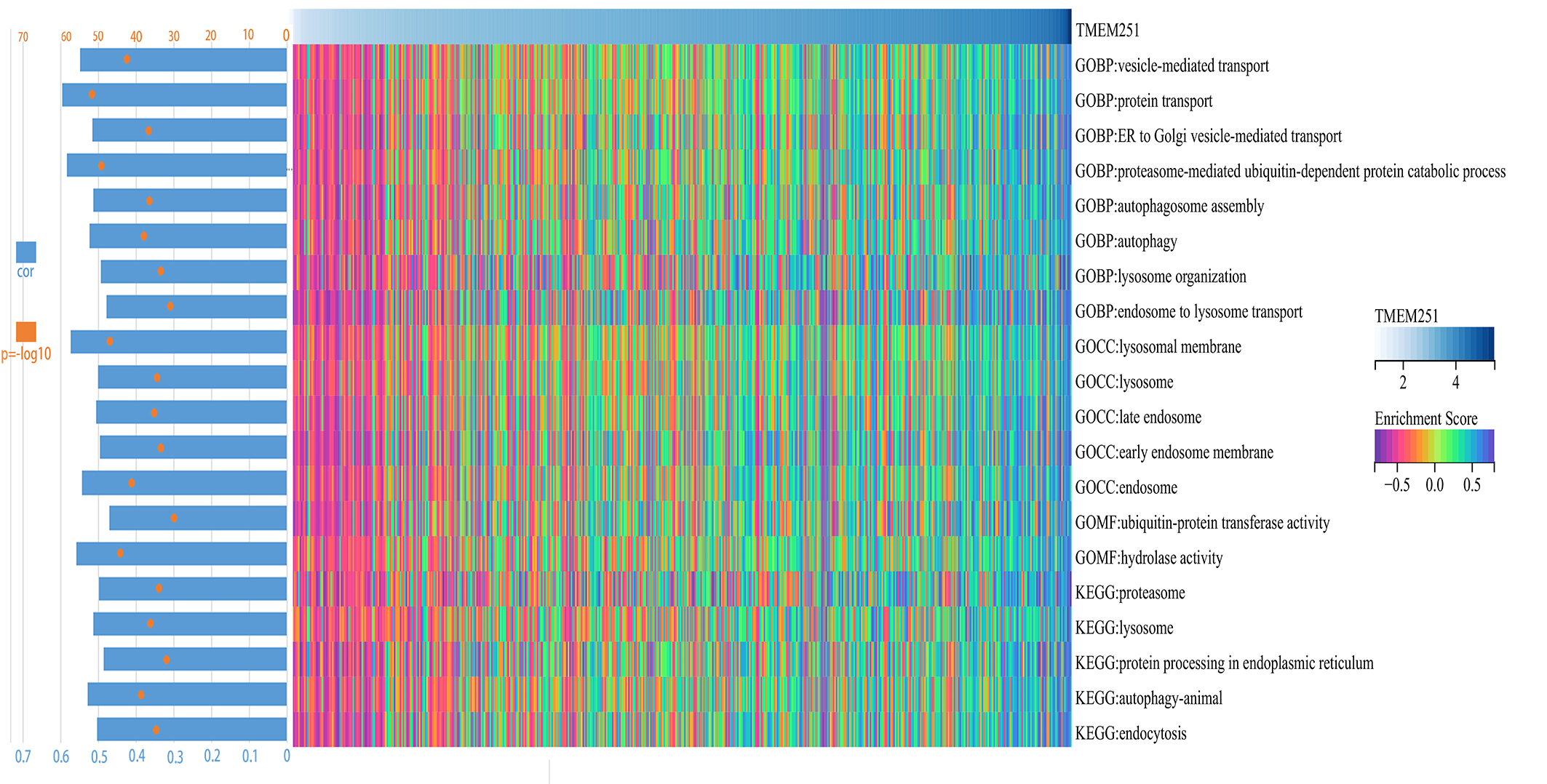

Supplement: Supplementary file 2 — Supplementary file2 (ZIP 28064 KB) [file 432_2023_5280_MOESM2_ESM.zip › Supplementary Figures/Supplementary Figure 2. Heatmap and correlation analysis between TMEM251 and enrichment scores.tif]

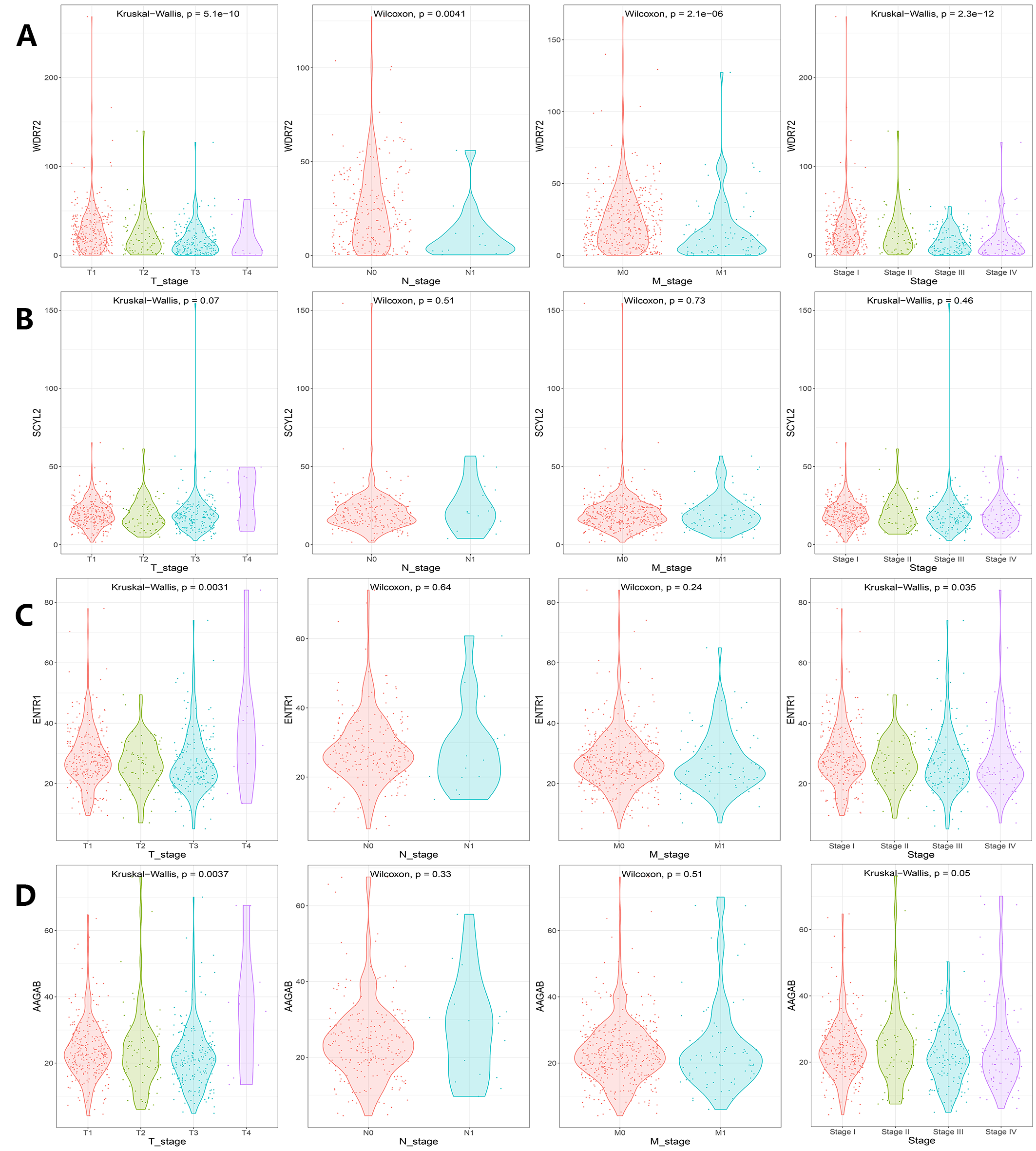

Supplement: Supplementary file 2 — Supplementary file2 (ZIP 28064 KB) [file 432_2023_5280_MOESM2_ESM.zip › Supplementary Figures/Supplementary Figure 3. Connections between hub genes and clinicopathological factors.tif]

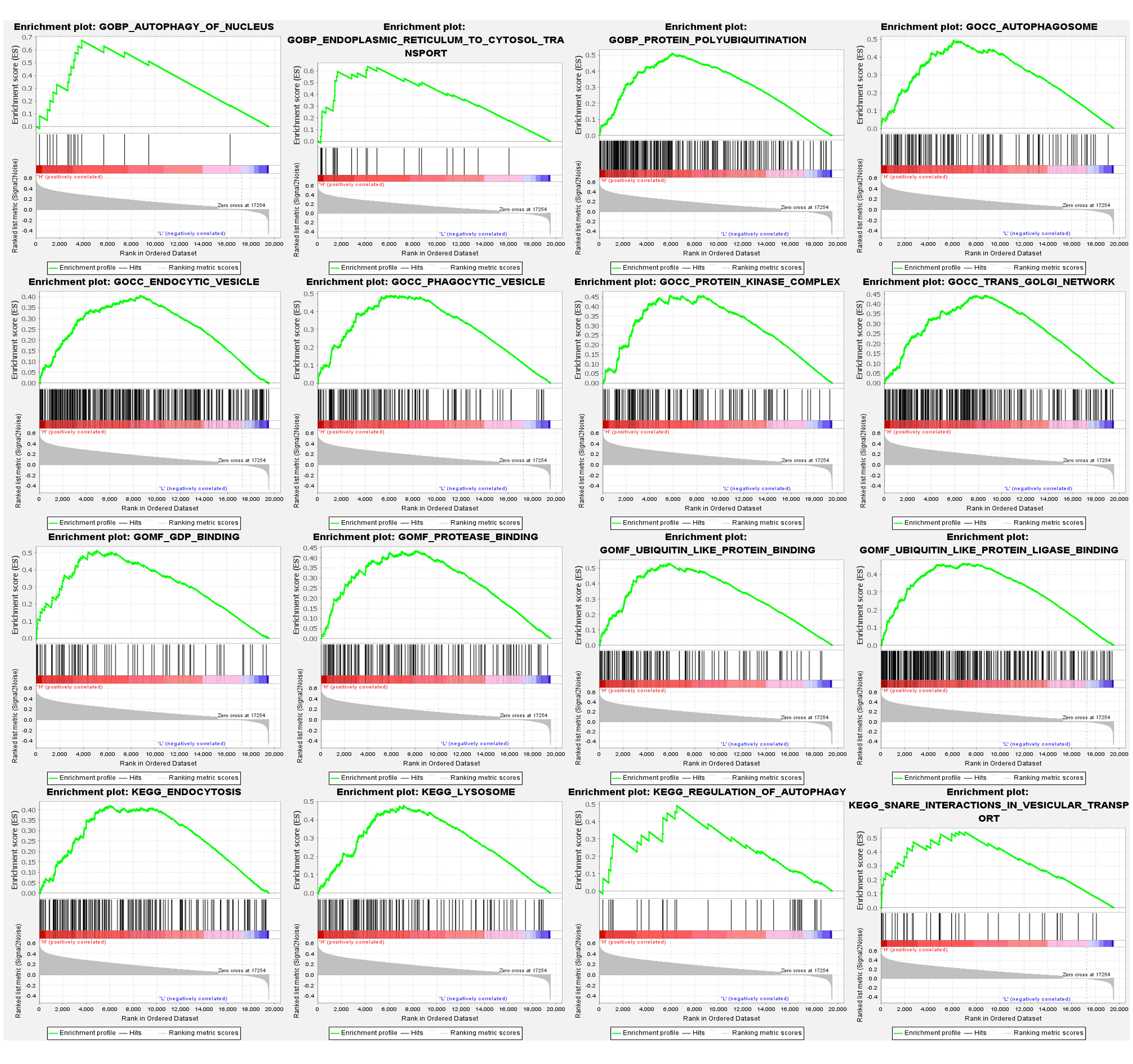

Supplement: Supplementary file 2 — Supplementary file2 (ZIP 28064 KB) [file 432_2023_5280_MOESM2_ESM.zip › Supplementary Figures/Supplementary Figure 4. Enriched AAMR-related gene sets in GSEA.tif]

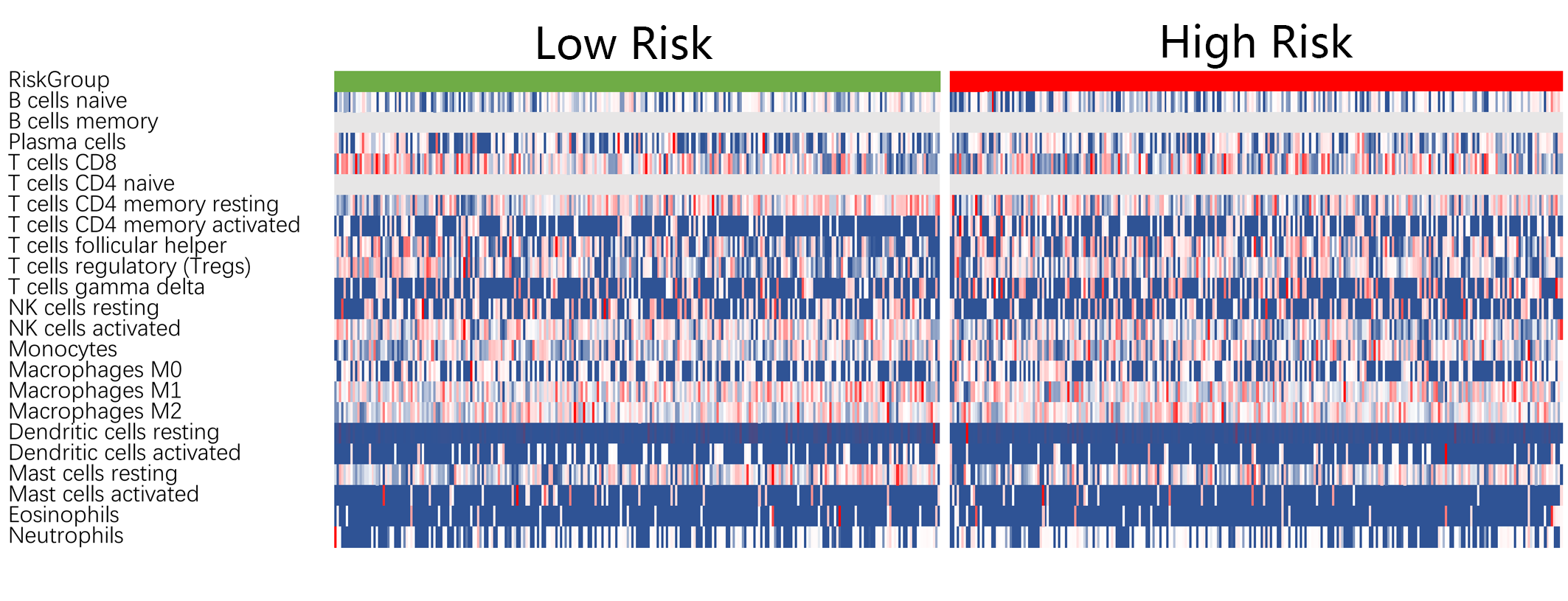

Supplement: Supplementary file 2 — Supplementary file2 (ZIP 28064 KB) [file 432_2023_5280_MOESM2_ESM.zip › Supplementary Figures/Supplementary Figure 5. Immune infiltration heatmap between different risk groups.tif]

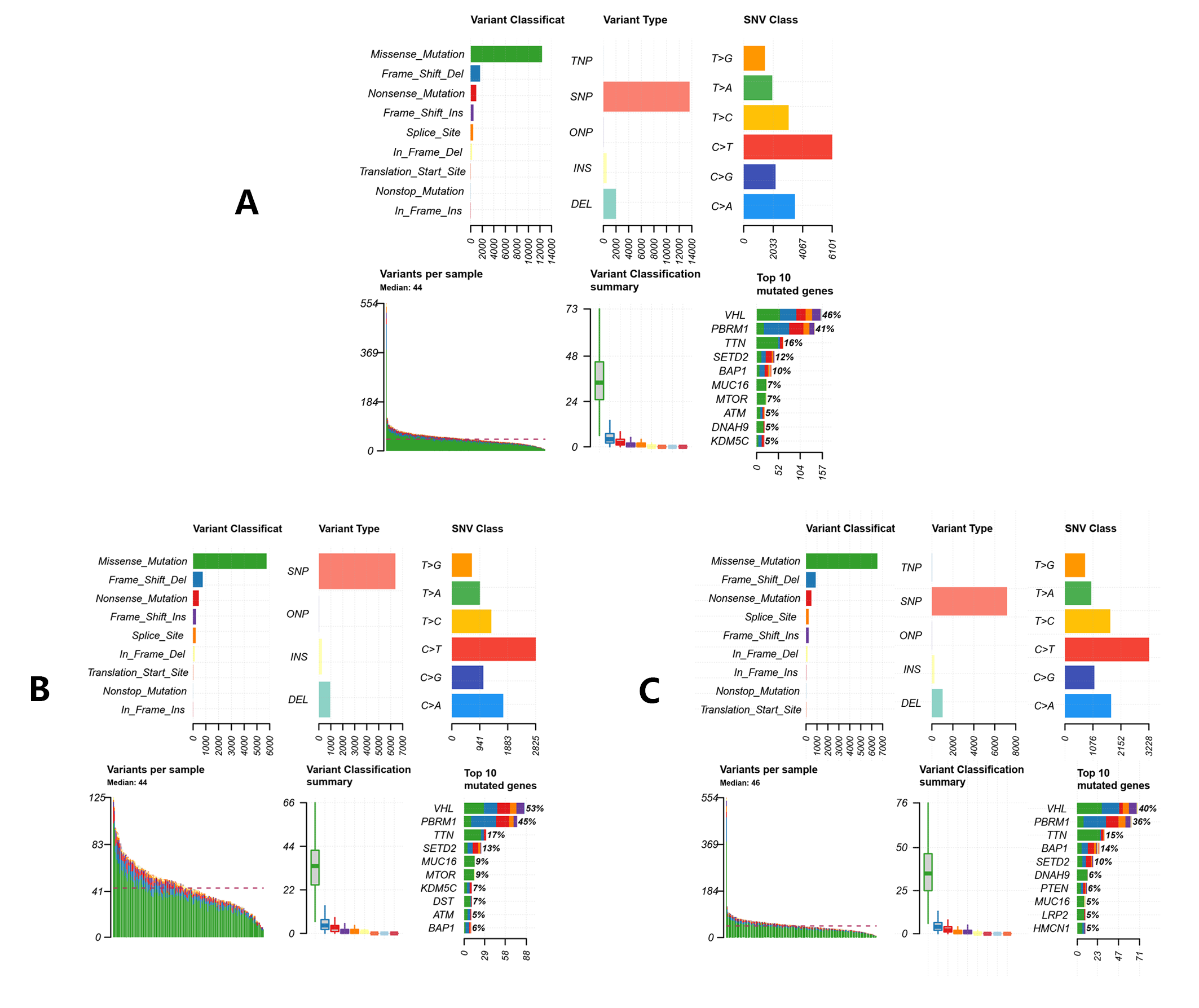

Supplement: Supplementary file 2 — Supplementary file2 (ZIP 28064 KB) [file 432_2023_5280_MOESM2_ESM.zip › Supplementary Figures/Supplementary Figure 6. Gene mutation information of samples.tif]
