# Supplementary material for: Functional enrichment analysis of LYSET and identification of related hub gene signatures as novel biomarkers to predict prognosis and immune infiltration status of clear cell renal cell carcinoma
Source: J Cancer Res Clin Oncol. 2023 Sep 23;149(18):16905–29. doi: 10.1007/s00432-023-05280-2 (PMC10645642; doi:10.1007/s00432-023-05280-2)
Supplement: Supplementary file 3 — Supplementary file3 (DOCX 20 KB) [file 432_2023_5280_MOESM3_ESM.docx]

Supplementary Material

Functional enrichment analysis of LYSET and identification of related hub gene signatures as novel biomarkers to predict prognosis and immune infiltration status of clear cell renal cell carcinoma

Yuxing Chen, Jinhang He, Tian Jin, Ye Zhang, Yunsheng Ou^*^

* Correspondence:

Yunsheng Ou

ouyunsheng2001@163.com

# Supplementary Data

**Data Sheet 1.** Gene Set Enrichment Analysis (GSEA) results between different risk groups.

# Supplementary Figures and Tables

## Supplementary Figures

**Supplementary Figure 1.** Protein-Protein Interaction Networks (PPI) of 310 genes from 20 AAMR-related gene sets.

**Supplementary Figure 2.** Heatmap and correlation analysis between TMEM251 and enrichment scores.

**Supplementary Figure 3.** Connections between hub genes and clinicopathological factors.

**Supplementary Figure 4.** Significantly enriched AAMR-related gene sets in GSEA.

**Supplementary Figure 5.** Immune infiltration heatmap of different risk groups.

**Supplementary Figure 6.** Gene mutation patterns of samples. **(A)** Gene mutation patterns of all samples (n=330). **(B)** Gene mutation patterns of the low-risk group. **(C)** Gene mutation patterns of the high-risk group.

## Supplementary Tables

**Supplementary Table 1.** Pearson correlation analysis between TMEM251 and other genes.

**Supplementary Table 2.** GO analysis results (BP).

**Supplementary Table 3.** GO analysis results (CC).

**Supplementary Table 4.** GO analysis results (MF).

**Supplementary Table 5.** KEGG analysis results.

**Supplementary Table 6.** 310 research genes from the 20 enriched gene sets.

**Supplementary Table 7.** Unsupervised cluster analysis based on 1234 TMEM251-coexpression genes.

**Supplementary Table 8.** Unsupervised cluster analysis based on 310 genes from the 20 enriched gene sets.

**Supplementary Table 9.** Unsupervised cluster analysis based on GSVA enrichment scores.

**Supplementary Table 10.** Differential gene expression analysis between cancer and normal samples.

**Supplementary Table 11.** The intersection of 310 genes and DEGs.

**Supplementary Table 12.** LASSO regression analysis of 55 research genes.

**Supplementary Table 13.** Stepwise multivariate Cox regression analysis for the predictive model.

**Supplementary Table 14.** Multivariate Cox proportional hazard regression model (without N stage).

**Supplementary Table 15.** Immune infiltration analysis (CIBERSORT) results.

**Supplementary Table 16.** Tumor mutation burden (TMB) scores of all samples.

**Supplementary Table 17.** Cell marker genes for single-cell sequencing analysis.
